# Application of Quaternized Chitosan in Enhancing Natural Organic Matter (NOM) Removal from Water by Flocculation

**DOI:** 10.1021/acs.langmuir.5c06027

**Published:** 2026-04-08

**Authors:** Mingyu Yuan, Heriberto Bustamante, Michael Gradzielski

**Affiliations:** † Stranski-Laboratorium für Physikalische und Theoretische Chemie, Institut für Chemie, 199914 Technische Universität Berlin, D-10623 Berlin, Germany; ‡ 72526 Sydney Water, Parramatta, NSW 2125, Australia

## Abstract

Humic acid (HA), which is a ubiquitous natural organic compound in surface waters to be removed during water treatment, can be removed by complexation with an oppositely charged polyelectrolyte. This process is typically done with commercial polycations such as poly­(diallyldimethylammonium chloride) (PDAD) but should equally be possible with bioderived polymers like quaternized chitosan (QCS), easily obtained with different degrees of quaternization by reaction with glycidyl trimethylammonium chloride (GTMAC). A comprehensive phase study at different pHs was conducted on solutions of HA and QCS of varying degrees of quaternization, which was compared to the behavior with PDAD. Complexation was monitored by ζ-potential measurements, HA flocculation, and floc growth by laser light diffraction, providing a deeper understanding of the flocculation mechanisms. The composition of the biphasic regions was determined by total organic carbon (TOC) measurements. Our findings reveal that the structural properties of polycations significantly influence the flocculation efficiency and that the charge density on the QCS and hydrophobic interactions play an important role in that process, as they shift the phase behavior as well as the kinetics of flocculation. In general, QCS performs similarly well in HA precipitation as PDAD and, in addition, its properties can become optimized by the degree of quaternization, where an intermediate value optimizes the properties in terms of HA removal efficiency and flocculation kinetics.

## Graphical abstract

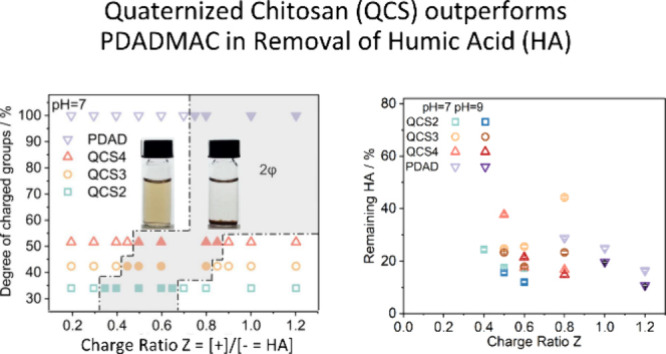

## Introduction

Ensuring access to clean drinking water remains one of humanity’s most pressing global challenges. However, the presence of natural organic matter (NOM) in surface water leads to significant challenges in water treatment. Conventional metallic coagulants are not sufficiently efficient in NOM removal presumably due to their complex chemical characteristics,^1^ which render it difficult to have a simple single compound being able to flocculate all of the contained molecules. NOM contributes to color, taste, and odor issues in urban water systems and, most importantly, forms disinfection byproducts (DBPs) during the disinfection process with chlorine (Cl_2_) for which there are strict regulatory limits, which are potential health hazards for public health.^2^

Polyelectrolytes are commonly used as secondary coagulants in standard industrial water treatment processes.^3^ Flocculation is a widely used conventional method for the removal of NOM in water treatment facilities, where humic acid (HA) constitutes a major component of NOM.^4^ HA is a complex mixture of organic macromolecules ranging from 400 Da to 300 kDa,^5^ contains a large number of carboxylic and phenolic groups,^6^ and therefore can be considered as a complex anionic polyelectrolyte. The interaction between HA and cationic polyelectrolyte (cPE) can be understood as the formation of a classical interpolyelectrolyte complex (IPEC), which is a concept well-established since the 1940s in colloid chemistry.^7,8^ For such systems involving oppositely charged polyelectrolytes, the primary driving force for complex formation is the resulting entropy gain due to the release of counterions and hydration water during the complexation process.^9^

According to the established understanding of the flocculation process, cPEs interact with negatively charged HA and reduce their electrostatic repulsion, thereby reducing their stability in aqueous solution and leading to the formation of larger aggregates and flocs.^10^ In addition, when the polymer chain of cPE is long enough, segments of the chain that dangle into the solution can adsorb onto neighboring particles, which is a process known as “bridging”.^11,12^ These adsorbed segments can then act as bridges, effectively linking particles together to form robust flocculates. This bridging effect enhances floc formation, leading to larger and more easily settling aggregates.

The conventional polymeric flocculants used in commercial water treatment are fully synthetic, including modified polyacrylamides (PAM),^13–15^ ammonium-based polycations^16–18^ and poly­(diallyldimethylammonium chloride) (PDAD),^19–21^ due to their high flocculation efficiency, low toxicity, and low cost. However, environmental concerns and the wish to turn to a more circular economy have prompted the search for more sustainable alternatives, such as chitosan^22^ or starch.^23^ Chitosan, as a biodegradable polymer, often derived from the shell of seafood, is increasingly considered in water treatment applications to address the environmental impacts associated with traditional chemical flocculants.^24,25^

One disadvantage of chitosan that hinders its general application in water treatment is its restricted solubility in the natural pH range, as it only becomes soluble at pH values below 6.5.^25^ Addressing this issue, various methods have been developed to enhance chitosan’s solubility by synthetic chemical methods.^26–28^ Grafting hydrophilic chains onto the chitosan backbone, as seen in PEGylated chitosan, not only improves solubility but also broadens its potential for applications in pharmaceutical formulations due to a more variable biological functionality.^29,30^ Another approach involves the introduction of quaternary ammonium groups. The simplest derivative is N,N,N-trimethyl chitosan (TMC), obtained via reaction with a methylating agent, e.g., methyl iodide.^31–35^ In addition, grafting a quaternary ammonium group as a side chain onto the chitosan backbone has also been widely applied. For example, water-soluble chitosan derivatives were achieved by initially reacting chitosan with ethyl acrylate, followed by further modification through substitution reactions involving aliphatic diamines or amino alkyl alcohols.^36^ Grafting 3-chloro-2-hydroxypropyltrimethylammonium chloride (CTA) has been reported to effectively improve the solubility of chitosan,^37,38^ and the success and extent of the modification can be determined by NMR.^39^ Conjugating glycidyl trimethylammonium chloride (GTMAC) onto chitosan chains introduces permanent cationic ammonium groups, which improve the polymer’s solubility and enhance electrostatic interactions with negatively charged contaminants, making these derivatives more effective in removing HA in water treatment.^35,40,41^ These modifications increase both its solubility across a broader pH range and its charge density, which are important parameters in the water treatment process.

Accordingly, quaternized chitosan (QCS) has been synthesized and advanced for different areas in the field of water remediation^41^ such as removal of bauxite from raw water.^42^ One interesting property of QCS is that, with its higher positive charge density, it typically exhibits enhanced antimicrobial properties compared to pure chitosan,^43^ where it has been observed that hydrophobizing in addition, by having one alkyl chain involved in the quaternization, further increases the antimicrobial properties substantially.^44^

In our study, to develop an environmentally friendly and potentially more efficient compound to remove HA, we explore the application of cationically modified chitosans, specifically QCSs, synthesized by conjugation with GTMAC,^40^ as cPEs in water treatment applications. By varying the degree of GTMAC substitution, modified chitosans with different charge densities were produced, and a systematic investigation of the phase behavior of mixtures of HA with a series of QCSs or poly­(diallyldimethylammonium chloride) (PDAD), as a fully charged linear reference polymer, was conducted. Additionally, the kinetics of flocculation was investigated by laser light diffraction (LLD), enabling real-time monitoring of HA floc formation. The aim of our study was to (i) systematically investigate HA–polyelectrolyte complexes under different pH and charge conditions, (ii) clarify the role of charge density and molecular architecture in governing the complexation behavior, and (iii) explore the possibility of replacing petro-polymer PDAD with biopolymer-based QCS. Such information will be helpful for developing more efficient flocculant materials for application in the water industry.

## Experimental Methods

### Materials

HA is Suwannee River Humic Acid Standard III purchased from International Humic Substances Society (IHSS).^45^ Acidic functional groups including carboxyl and phenolic groups were regarded as the charged groups at a certain pH range; for details regarding the characterization, see the SI.

150 kDa PDAD (low molecular weight, 100–200 kDa, corresponding to a stretched length of 310–620 nm) was purchased from Sigma-Aldrich as 20 wt % solutions in water and freeze-dried before usage.

Chitosan (low molecular weight, 50–190 kDa, corresponding to a stretched length of 155–580 nm) was purchased from Sigma-Aldrich. To remove any possible residual chloride ions from its production, chitosan was precipitated by pH tuning, washed, and freeze-dried before use. The degree of deacetylation (DDA) was determined by potentiometric titration (Figure S2). All samples were prepared with Milli-Q water (18.2 MΩ cm at 25 °C).

For all samples, the HA concentration was 40 mg/L, which corresponds to a nominal charge concentration of 0.157 mM at pH 9, as calculated from the averaged molecular weight of the charged unit determined from titration (*M*_w_(charge) = 255 g/mol; see the titration curve in Figure S1). The pH of HA solutions was adjusted to 7.0 and 9.0 with 0.1 M NaOH solutions before the addition of cPEs (PDAD or chitosan).

The cPE concentration was varied to achieve a specific nominal charge ratio *Z*, which is defined as *Z* = [+]/[−] = [quaternized amino groups of chitosan]/[carboxylic and phenolic groups of HA]. This in turn means that this is a nominal charge ratio, but at pH 9, it should also be close to the actual charge ratio (as here only the phenols are not ionized to a larger extent). Of course, it has to be noted that the real charge of the macromolecules depends on the pH, where at lower pH the HA becomes more neutralized and the free amino groups of the chitosan become increasingly protonated, which means that the real *Z* value depends on the pH. In addition to pH, the charge state will also depend on the mixing ratio of both components due to their mutual interaction in the formed complexes, which modifies the acidity/basicity of their functional groups.

### Quaternization of Chitosan

Water-soluble quaternary ammonium chitosan (QCS) derivatives were synthesized by grafting GTMAC onto the glucosamine residues of chitosan polymers using a previously established method^46^ (for details, see SI). The mole ratio of GTMAC to chitosan was varied from 2:1 to 4:1 to produce QCS with different degrees of substitution (DS) ranging from 34.0 to 51.6%, as characterized by conductometric titration with AgNO_3_ (Figure S3). Based on the GTMAC to chitosan molar ratios, the QCSs were designated as QCS2, QCS3, and QCS4 and contained 34.0, 42.4, and 51.6 mol % quaternization. Further details are given in Table 1.

**Table 1:**
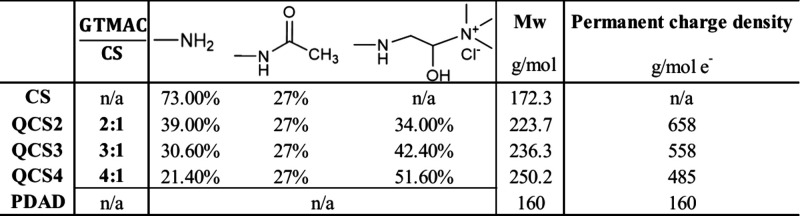
Composition, Molar Feed Ratio GTMAC/CS^a^, Ratios of the Functional Groups, Molecular Weights, and Charge Densities for the Different Samples of QCSs and PDAD

a In GTMAC/CS, CS refers to the moles of monomer units.

### Confocal Laser Scanning Microscopy

Confocal laser scanning microscopy (CLSM) images of precipitated flocs were obtained with a Leica CLSM system in a reflectance configuration using a 488 nm laser. Complexes formed between HA and various cPEs were carefully transferred to an 8-well chamber slide immediately after mixing and left undisturbed for 24 h. Subsequently, they were directly examined under CLSM to avoid any disruption to the floc structures. This method ensured minimal disturbance, allowing for accurate imaging and analysis of the floc formations. For better visualization, a lookup table (LUT) was applied to transform color input values to alter the color of images.

### ζ-Potential

ζ-Potential measurements were carried out using a Litesizer 500 (Anton Paar) at 298 K, employing a laser with a wavelength of 658 nm. The ζ-potential was determined via electrophoretic light scattering (ELS), which provided the electrophoretic mobility *U*_E_. The ζ-potential was calculated using the following relation:

$$\zeta =\frac{3\eta U_{E}}{2\varepsilon f(\kappa \alpha )}$$ 1

where η is the dynamic viscosity, *U*_E_ is the electrophoretic mobility, ε is the dielectric constant, and *f*(κα) is the Debye factor. For particles dispersed in aqueous media and significantly larger than the Debye screening length, *f*(κα) was approximated as 1.5, according to the Smoluchowski approximation.

### UV Absorbance

UV absorbance measurements of HA solutions were conducted using a Cary 50 UV–vis spectrometer equipped with a 1 cm path length quartz cuvette. Specifically, the absorbance at 254 nm (UV254) was measured to calibrate HA concentrations within the range of 0.25–25 mg/L (Reference). A strong linear correlation was observed between UV254 absorbance and HA concentration, described by the equation *y* = 0.02711*x* – 0.00364 (*R* ^2^ = 0.999), where *y* represents the absorbance at 254 nm and *x* is the HA concentration in mg/L. This calibration allows for reliable quantification of HA in unknown samples. The percentage of remaining HA after treatment can then be calculated using the following equation:

$$\begin{array}{c} \mathrm{Remaining}\;\mathrm{HA}(\%)=\left(\frac{C}{C_{0}}\right)\times 100\% \end{array}$$ 2

where *C*_0_ is the initial HA concentration (40 mg/L in our work), and *C* is the residual concentration in the supernatant after precipitation, determined from the measured UV254 absorbance.

### Laser Light Diffraction

LLD was utilized to monitor the development and growth of the HA-cPE complex size during the flocculation process. A wet dispersing system CUVETTE was integrated with a laser diffraction sensor HELOS (Sympatec) for dispersion. The measurement was conducted in a 50 mL cuvette with a stirring rate of 600 rpm to achieve adequate mixing. An R5 lens with a measurable particle size range of 0.5/4.5–875 μm was selected to evaluate the particle size. Time-sliced measurements over 10 min with a resolution of 10 s were performed after the mixing of HA solution and cPEs for time-resolved investigation of the flocculation process. The temperature was 25 °C, and as time 0, we took the moment of first mixing, which means that our actual LLD measurement only began about 30 s after the mixing of the solutions, as that was the time required for transferring and diluting the samples.

For the laser diffraction measurements, the optical concentration of the sample is a critical parameter that must be precisely controlled to around 20% to obtain reliable data. The optical concentration is a measure of obscuration of the center of the laser detector caused by the particles in the laser beam. It is calculated using the formula:

$$\begin{array}{c} C_{\mathrm{opt}}=\frac{I_{\mathrm{ref}}-I_{\mathrm{mes}}}{I_{\mathrm{ref}}} \end{array}$$ 3

where *C*_opt_ is the optical concentration, *I*_ref_ is the mean intensity in the detector center during the reference measurement (as a reference, we employed Milli-Q water (18.2 MΩ cm at 25 °C)), and *I*_mes_ is the mean intensity in the detector center during the measurement. Accordingly, for the measurement, we had to adapt the HA concentration to meet this condition.

## Results and Discussion

### Phase Diagram

As a first step of our investigation, we thoroughly studied the macroscopic phase behavior of the HA-cPE complexes at 25 °C and for a fixed HA concentration of 40 mg/L, which is somewhat above the typical concentration of HA in raw water but allows for better experimental observation. The polyelectrolyte concentration was varied to achieve a specific nominal charge ratio *Z* ranging from 0 to 1.2, and samples were studied at pH 7 and 9. The macroscopic phase behavior of these aqueous solutions for these two pH conditions can be seen in Figure 1. For each sample set, a transition from a monophasic to a biphasic region can be seen, indicating the formation of insoluble complexes as positively charged polyelectrolyte molecules complex with negatively charged HA molecules.

**Figure 1:**
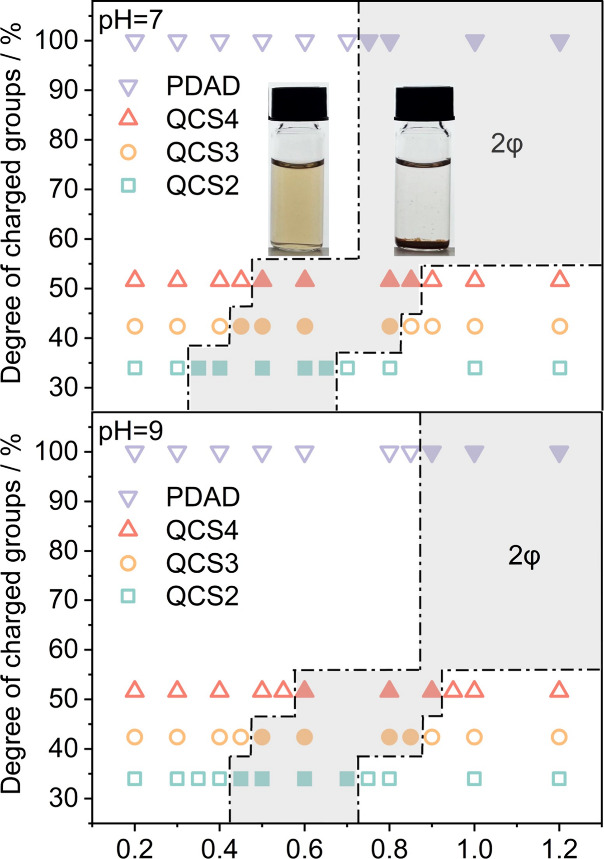
Phase diagram of 40 mg/L HA and different added polyelectrolytes, the added amount being characterized by the nominal charge ratio *Z* (= [+]/[−]) at pH 7 and 9, respectively (*T* = 25 °C). The different polyelectrolytes are classified on the *y*-axis according to their percentage of (nominal) charges per monomer unit. Open symbols refer to monophasic regions, while full symbols refer to the biphasic region.

On further increasing the PE dosage, the complexes can be restabilized, thus switching back to the clear solution with water-like viscosity, same as the monophasic region for a lower charge ratio value. However, a noteworthy observation is that once precipitates have formed in the biphasic region, they do not dissolve upon subsequent addition of further cPEs. This suggests that the precipitates exhibit considerable stability and their redispersion is kinetically constrained.

Two pH values of 7.0 and 9.0 were chosen, as they are relevant to the practical situation in conventional water plants. At pH 9, the carboxylic groups are fully dissociated, and the phenolic groups are partially dissociated. At the same time, the protonation of the amino group of chitosan is negligible, i.e., the positive charge can be fully attributed to the permanent charge of substituted GTMAC molecules. This means that at pH 9, the nominal charge ratio of *Z* corresponds well to the real charge ratio. In contrast, at pH 7, the degree of deprotonation for HA was decreased by around 20% compared to that at pH 9, as calculated from the titration curve of IHSS HA in Figure S1, which means that the tendency of HA to interact electrostatically will be somewhat reduced. At the same time, almost 20% of the amino groups from modified chitosan will be protonated (as calculated from the titration curve of unmodified chitosan in Figure S2), thus adding additional positive charges to the system. This means that the real charge ratio at pH 7 is larger than the value of *Z,* and for a comparison, the phase diagram of Figure 1 is also shown in Figure S4 for the more realistic charge ratio, as calculated based on the charging deduced from the titration curves of the individual components (and neglecting the fact that the state of ionization will further change due to the complexation; a plot of how the real charge ratio and the nominal charge ratio *Z* are related at pH 7 is shown in Figure S5 for the three different QCSs). Unless otherwise specified, all charge ratios in the paper refer to the nominal charge ratio (which is experimentally solidly defined), allowing consistent comparison between pH 7 and pH 9 at identical dosages.

As a result, at pH 7, effective phase separation takes place at lower PE dosages (as the real *Z* would be larger, and if one takes that into account, both phase diagrams look more similar; see Figure S4). For example, the HA-PDAD system exhibits the transition from the monophasic to the biphasic region at *Z* = 0.75 and 0.90, respectively, at pH values of 7 and 9, but the corrected *Z* value becomes 0.91 at pH 7. Also, at pH 7 a biphasic region in the charge ratio range of 0.35–0.65 can be observed for the HA-QCS2 system, while at pH 9, it slightly shifts to a larger charge ratio of 0.45–0.7. However, it is important to recognize that even after adjusting for the real charge ratio, the converted results at pH 7 do not align exactly with the phase diagram observed at pH 9. Interestingly, at pH 7, a higher real *Z* value is required for observing precipitation, which indicates that having the HA as deprotonated as possible is important for effective precipitation. In general, this means that complexation and flocculation are not just determined by the charge ratio, but other parameters such as molecular configuration and solubility under various environmental conditions play an important role.

Comparing the different cPEs in Figure 1, one observes that PDAD, with its 100% charging, precipitates near the nominal charge neutralization point where the charge ratio *Z* = 1 at pH 9. The onset of precipitation for pH 9 is at *Z* = 0.9, while for pH 7 the onset is found to be *Z* = 0.7, as discussed before; this shift can be explained by the effectively different degree of protonation of HA that is pH-dependent. In contrast, modified chitosan, with its degree of permanently charged groups ranging from 34 to 52%, shows precipitation much before it reaches the nominal stoichiometric charge neutralization point. This phenomenon may primarily be attributed to the much higher flexibility of the charged group of QCS, compared to PDAD, where the charge is fixed to the polymer backbone (see Figure 2). This structural property should facilitate effective electrostatic interaction with the charged groups of the HA, which are largely fixed within their aromatic structure and therefore not very flexible themselves. In addition, at pH 7, a percentage of 20% of the amino groups should be charged and still further ionization may take place due to complex formation (which lowers the p*K*_a_ of the protonated amino groups within the formed complexes, due to the vicinity of the negative charges of the HA).

**Figure 2:**
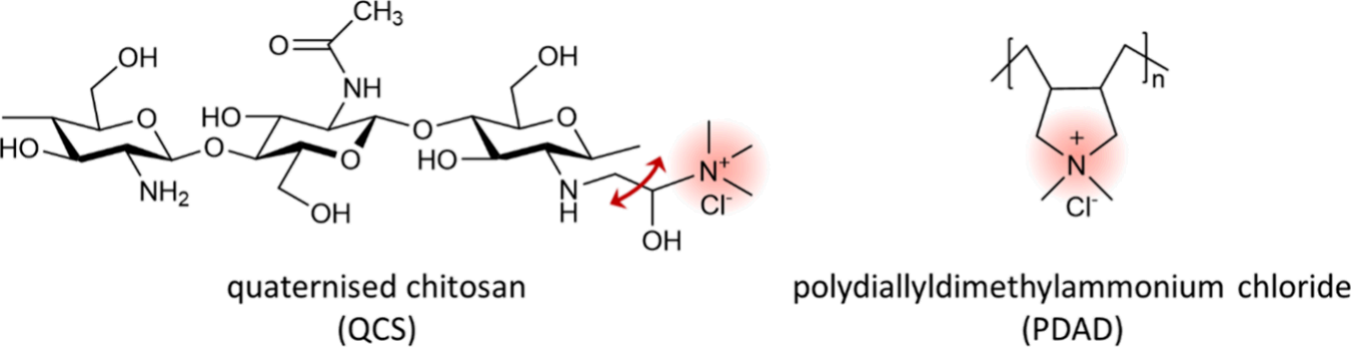
Structures of QCS and PDAD. The charged regions are highlighted.

In addition, one has a higher intrinsic hydrophobicity of the chitosan backbone, and the QCS may interact more effectively with the hydrophobic components of HA molecules, thereby amplifying the heterocoagulation mechanism. The significance of hydrophobic interactions is further illustrated by comparing the different QCSs. A systematic shift of the phase behavior is seen, and for QCS2, which possesses the lowest degree of charged groups and consequently the highest hydrophobic character for a given charge ratio value, the onset of precipitation is shifted the most to the left in the phase diagram, where the transition from the monophasic to the biphasic occurs at *Z* = 0.35 for pH 7, while for QCS3 and QCS4, it occurs at 0.45 and 0.50, respectively. This shift shows the critical role of hydrophobic interactions (and potentially interactions with the amino groups), which occur in synergy with the charge neutralization mechanism, underscoring the complex interplay between hydrophobic and electrostatic forces in achieving effective coagulation as well as enhancing the phase separation. However, when plotting the phase diagram over the mass concentration of added polyelectrolytes, as illustrated in Figure S6, QCSs exhibit a biphasic region at a higher concentration compared to PDAD due to its relatively lower charge density. When comparing different QCSs, the relationship between the degree of GTMAC substitution and the phase behavior does not follow a linear pattern. Specifically, QCS3, which has a median degree of GTMAC substitution, unexpectedly exhibits the highest concentration range for the biphasic region among the QCSs.

Furthermore, to visualize the interaction of HA and modified chitosans with respect to their ability of floc formation for varying degrees of substitution and for different pH conditions, confocal microscopy was employed to observe the morphology of flocs formed in the biphasic region. Examples of flocs formed at pH 7 and 9 by the different QCSs with HA at charge ratio *Z* = 0.5 are shown in Figure 3, along with HA-PDAD flocs at charge ratio *Z* = 1.0 (as there are no flocs formed at *Z* = 0.5). For QCS2 with the lowest degree of substitution of GTMAC, and thus the lowest charge density, a glossy and more homogeneous appearance and less distinct floc formation can be seen. In contrast, QCS4, which has the highest degree of substitution, leads to the formation of clearer and more densely packed flocs under both pH conditions. The generally denser floc formation at pH 7, as compared to pH 9, also indicates the favorable rearrangement of HA complexes in a more acidic environment. The results suggest that the degree of substitution not only influences the phase behavior but also affects the spatial packing density of flocs, which has potential implications for the floc strength, a parameter that requires precise control in the practical water treatment process to achieve efficient filtration.^47^ Interestingly, for PDAD, no significant effect of the pH is seen, and always rather filamented flocs are formed. The independence from pH may simply be explained by the fact that its charge state does not change, and therefore, its extent of interaction with HA also varies little.

**Figure 3:**
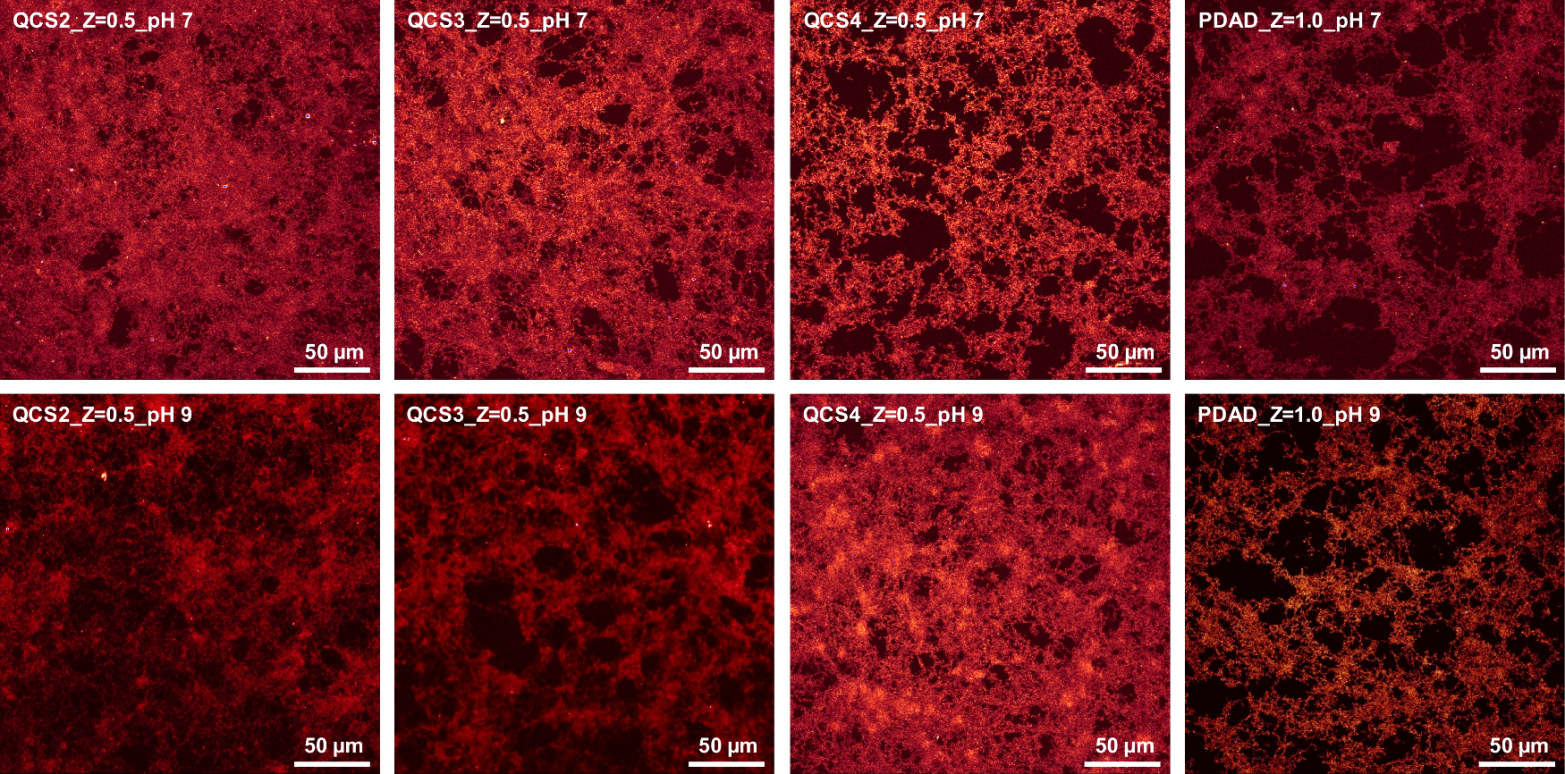
CLSM images of precipitated flocs at *Z* = 0.5 for the different QCSs with HA and for *Z* = 1.0 for PDAD with HA (size bar: 50 μm).

### Stability of HA Complexes: ζ-Potential

The colloidal stability can be estimated by the ζ-potential, which is the potential difference between the dispersion medium and stationary shear plane of the HA complexes. It has been used for a long time in water treatment facilities to determine colloidal stability and to optimize coagulant dosage.^21,48^ The values of the ζ-potential of HA complexes with different cPEs are shown in Figure 4 and given in Table S1 in the Supporting Information. One observes a consistent increase of the ζ-potential for all cationic PEs added to the system. A higher value is always observed for pH 7, which is consistent with incomplete deprotonation of HA molecules and the partial protonation of amino groups of modified chitosans. However, when compared for the real charge ratio, the curves for pH 7 and 9 look rather similar (see Figure S7).

**Figure 4:**
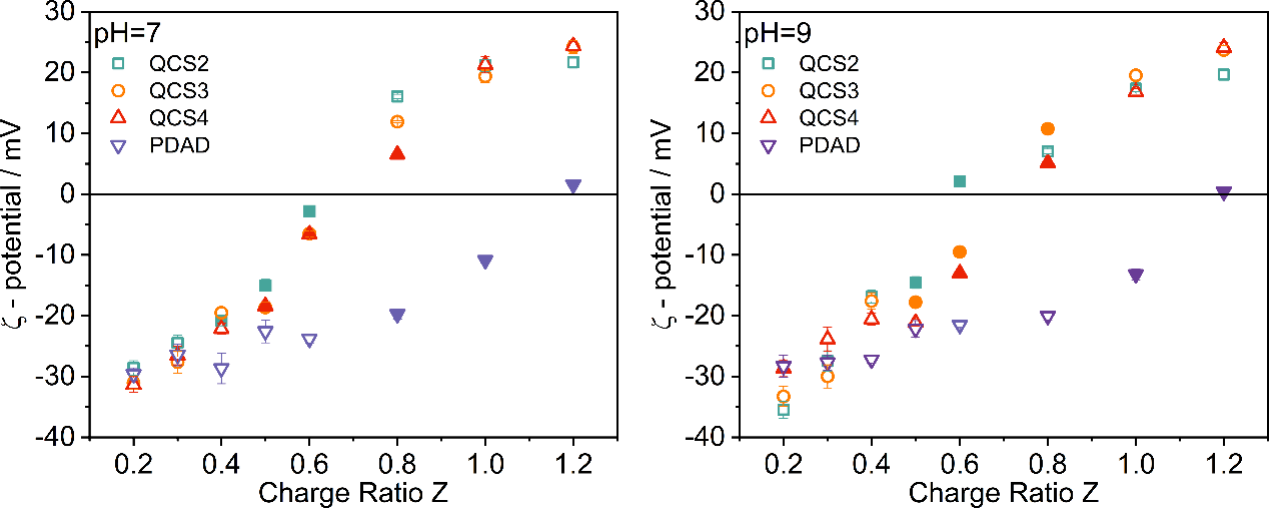
ζ-Potential for complexes of HA and polyelectrolytes at different charge ratio *Z* values at pH 7 and pH 9, respectively (measurements performed at 25 °C; full symbols denote samples located in the biphasic region, but measurements were performed before precipitation set in). Error bars are given or are smaller than the symbol size.

The values of the ζ-potential of modified chitosan with various charge densities are rather similar, whereas those in the reference PDAD system are much lower, confirming that the flexibility of the charged group on the QCS backbone allows for more effective neutralization of the negative charges on HA molecules, which leads to a larger shift in the ζ-potential at lower polyelectrolyte addition. In addition, the ability of chitosan to form hydrogen bonds and to complex via hydrophobic interactions potentially facilitates the formation of more stable HA complexes, effectively increasing the ζ-potential. In any case, it is a very interesting observation that charge neutralization takes place for much lower nominal charge compensation in the case of QCS compared to PDAD.

### Removal Efficiency of HA

HA is known to absorb strongly in a certain UV range due to its conjugated aromatic rings, which leads to the utilization of the absorbance at 254 nm (UV254) as a water quality test parameter that provides a quick measurement of the HA content in water.^49,50^ As illustrated by the UV–vis spectra of HA solutions of different concentrations and the corresponding calibration curve in Figure S8 of the Supporting Information, a linear relationship between the HA concentration and UV254 value can be derived to quantify the remaining HA after treatment for those biphasic sample sets, as seen in Table S2.

In Figure S9, the relationship between UV254 values of biphasic HA-PE systems 24 h after mixing and charge ratio *Z* is shown. The absorbance value can be converted to the concentration of the remaining HA, which is shown in Figure 5. Corresponding to the macroscopic phase behavior described before (Figure 1), in the range of charge ratios *Z* from 0.8 to 1.2, a huge drop in the UV absorbance at 254 nm can be seen for HA-PDAD; however, for the HA-QCS systems, such a phase separation occurs at lower charge ratio values, ranging from 0.4 to 0.8. It is observed that all QCS derivatives demonstrate similar removal efficiencies, albeit with a slight shift corresponding to the phase behavior. In addition, the removal efficiency increases somewhat with decreasing degree of quaternization, i.e., in the row from QCS4 over QCS3 to QCS2. Apparently, a higher degree of hydrophobicity and lower charge density help in quantitative HA precipitation, becoming more efficient, in agreement with the flocs observed in Figure 4. In absolute values, the removal efficiency of PDAD is similar to that of QCS. Notably, the removal efficiency of all cPEs is enhanced somewhat at pH 9 in comparison to that at pH 7. This increase in efficiency at a higher pH can be attributed to the higher degree of deprotonation of HA molecules, leading to stronger electrostatic interactions with the positively charged polyelectrolytes, thereby improving the aggregation and subsequent removal efficiency of HA.

**Figure 5:**
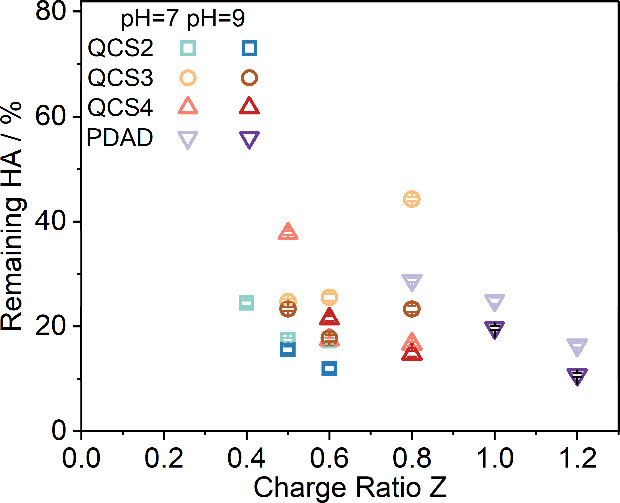
Remaining percentage of HA in the supernatant 24 h after mixing HA and various polyelectrolytes under pH 7 and pH 9, respectively, as determined by UV254 spectroscopy at different nominal charge ratios of *Z* at 25 °C. Error bars are given or are smaller than the symbol size.

### Fluorescence Probe Studies

After having considered so far mostly the macroscopic behavior of the HA/polycation systems, we were now also interested in the question of the extent to which hydrophobic domains may be formed within the complexes. To address this question, we employed 6-propionyl-2-(dimethylamino)­naphthalene (Prodan) as a neutral solvatochromic probe to study the local aggregation behavior and polarity, as Prodan reactivity is sensitive to its microenvironmental polarity.^51,52^ Accordingly, one can for instance study the aggregation behavior of LCST block copolymers by fluorescence spectroscopy with Prodan as a polarity probe, via changes in the fluorescence intensity and peak position,^53^ where a blue shift is observed with an increasingly nonpolar microenvironment.^51,52^ The changes of fluorescence intensity are less obvious but show a tendency to be higher for a more hydrophobic microenvironment but will, of course, also be strongly affected by quenching effects.^54^

Figure S10 shows the fluorescence emission spectra of 1 μM Prodan probe in the presence of varying concentrations of HA at pH values of 7 and 9, and the corresponding extracted wavelength of the maximum emission (λ_max_) and emission intensity (*F*_max_) are shown in Figure 6. A slight shift of the spectra maximum λ_max_ to lower wavelengths with increasing HA concentration is observed mainly for pH 9, indicating the presence of weakly hydrophobic domains within the HA aggregates at higher concentration. For both pH values, a substantial decrease of fluorescence intensity of the Prodan signal is seen with the addition of very small amounts of HA, likely due to quenching. The fluorescence intensity at pH 7 is generally higher than that at pH 9, especially for higher HA concentrations, indicating a more hydrophilic environment at pH 9 as more carboxylic and phenolic groups within HA are deprotonated. In addition, as shown in Figure S11, the fluorescence spectrum of HA at 40 mg/L shows a broad peak around 450 nm, which is independent of pH. Apparently, the intrinsic optical properties of HA are not affected by pH, while Prodan senses here a somewhat different microenvironment.

**Figure 6:**
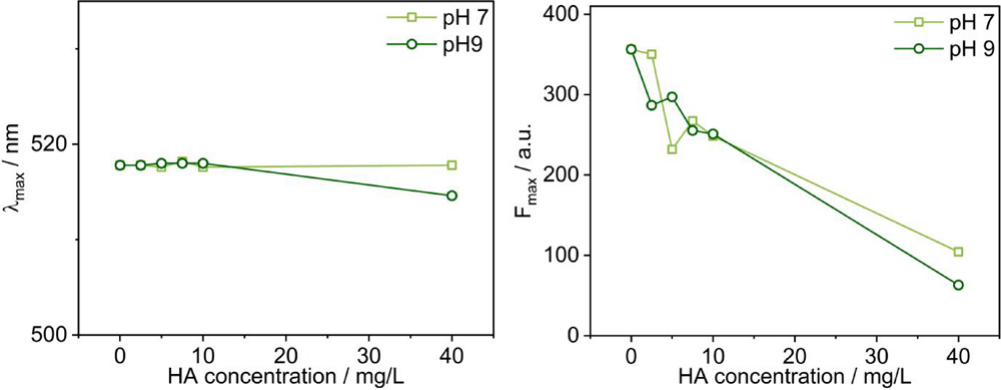
Extracted wavelength of the maximum emission (λ_max_) and its emission intensity (*F*_max_) of the polarity-sensitive 1 μM Prodan probe in varying concentrations of HA, at pH 7 and pH 9, respectively.

For the investigation of the local polarity in the complexes of HA with various cPEs, Prodan was added to HA-cPE complex solutions with different *Z* values, and the emission spectra obtained are shown in Figures S12 and S13. Figure 7 shows the extracted wavelength of the maximum emission (λ_max_) and its emission intensity (*F*_max_). All samples were measured immediately after mixing, i.e., before macroscopic phase separation could set in.

**Figure 7:**
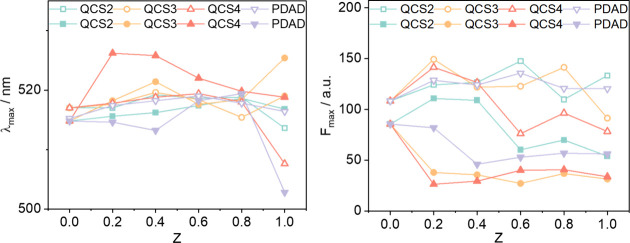
Extracted wavelength of the maximum emission (λ_max_) and its emission intensity (*F*_max_) of the polarity-sensitive 1 μM Prodan probe as a function of the nominal charge ratio *Z* for a constant HA concentration of 40 mg/L, at pH 7 (open symbols) and pH 9 (full symbols), respectively.

Starting from the values seen for pure 40 mg/L HA solutions (*Z* = 0), the addition of all of the different cPEs to HA solutions leads generally only to small changes of the peak position (λ_max_), whereas the peak intensity (*F*_max_) shows more marked changes (Figures S12 and S13). This indicates that the polarity of the microenvironment of Prodan changes only rather little upon HA complexation by cPEs, i.e., apparently no marked formation of hydrophobic domains takes place, even under flocculation conditions. The decrease in intensity (*F*_max_), as in the case of the pure HA, can be ascribed to a quenching effect due to the presence of the HA. In general, the changes of λ_max_ and *F*_max_ are more pronounced at pH 9.

Looking in more detail at the data, for PDAD, only very small changes are observed for λ_max_ and only at pH 9 *F*_max_ decreases substantially. The behavior is rather similar for QCS2 and QCS3, indicating that the Prodan encounters at best a rather small change of its microenvironment due to the complexation with the polycation. Clearly different is the behavior for QCS4 with its highest degree of quaternization of 51.6%. Here, the shift of the peak to ∼525 nm suggests even an increase in polarity upon addition of QCS4, which has to be attributed to the high charge density introduced by QCS4, which apparently locally leads to a more polar environment of Prodan. The emission intensity (*F*_max_) for all complexes at pH 9 was lower compared to those at pH 7 and drops strongly upon complexation with the polycation (here only for QCS2 a quite different behavior is seen, whose origin still remains unclear), thereby confirming the presence of a more polar microenvironment of Prodan. This aligns with findings from pure HA solution at pH 9 and may be attributed to the higher degree of charging of HA at pH 9. Such Prodan quenching can be attributed to the more extensive interaction within the complexes, where a more compact structure is formed that potentially limits the mobility of Prodan.^55,56^ The plateau at a higher charge ratio *Z* suggests that once HA molecules are somewhat complexed with polycations (up to *Z* ∼ 0.4–0.6), further addition of polycations does not change the microenvironment any more. Similar phenomena have been observed in previous research, studying the interactions between Prodan and HA in the presence of cations Na^+^, Ca^2+^, and Mg^2+^, where cation concentrations below the HA charge density significantly reduce Prodan quenching, while higher concentrations have no significant further effect.^57^ In contrast, the changes seen for the different cPEs at pH 7 are rather small, and apparently here no formation of a different microenvironment takes place.

### Flocculation of HA Complexes via Light Diffraction

To monitor the flocculation processes of HA with the different polycations, time-slice LLD experiments were performed. As mentioned in the methods part for achieving the appropriate optical concentration, direct measurement of systems with 40 mg/L HA often results in optical concentrations that exceed the instrument’s measurement range, leading to inaccurate data and risk of damaging the instrument. To circumvent this problem, we initially mixed HA with various polycations to form preliminary structures at a controlled concentration of 40 mg/L for 30 s. Subsequently, the mixture was quickly diluted with water to a final concentration of 10 mg/L HA. This step was performed to ensure that the flocculation process fell within the detectable range for subsequent measurements. This approach then mitigates the issues associated with high or low optical concentrations (and is also closer to realistic conditions during the water treatment process).

Figures S14 and S15 show the obtained particle size distribution curves recorded for HA complexes formed by various QCSs with a charge ratio *Z* of 0.4 at pH 7 and a charge ratio *Z* of 0.5 at pH 9, respectively, and for comparison, identical experiments were performed with PDAD at *Z* = 1.0. The observed temporal evolution of particle size distribution was systematically reproduced to ensure the reliability and consistency of our results. For all of the samples, the intermediate stage is characterized by a roughly exponential growth in the particle size *x*(50%) and leveling off to a final value within the time frame of this experiment. One observes a state of quasi-stability during this phase, indicative of a slowed or attenuated growth rate. Only for the HA complexes formed by QCS2 with a charge ratio *Z* of 0.4 at pH 7, a rather large size is seen already for the first data point, indicating that substantial growth has already occurred before for this least charged of the QCS polymers.

For a quantitative analysis, the change of particle size over time was further empirically examined using a logistic growth model (see SI for details),^58^ as shown in Figure 8. This model, for which the initial growth stage is approximately exponential, followed by a slower process and ending with a plateau indicating the saturation state, is fully consistent with the experimental observation of the particle size development over time. All of the parameters derived from this analysis are presented in Table S1. For the fastest evolving QCS2, one finds the inflection point already at 41.89 s. HA-QCS3 complexes with the same charge ratio *Z* of 0.4 at pH 7 displayed a slightly lower final value of *x*(50%) of 34.2 μm, with a higher growth rate of 0.017 s^–1^. The inflection point at 136.9 s for this set indicates that the midpoint of growth occurred well within our observation period, allowing for a complete capture of the growth kinetics from initiation to near saturation. Switching to QCS4 with the highest degree of GTMAC substitution, this sample set presented the smallest final *x*(50%) value of 30.5 μm among the three, yet it exhibited the fastest growth rate of 0.020 s^–1^ and the latest inflection point of 171.4 s, which reflects a slower initial growth phase followed by a more rapid growth around the midpoint of the process. Effectively this system shows an incubation time for the particle growth, which to a lesser extent is also seen for QCS3. QCSs with higher solubility tend to exhibit a longer inflection time, indicating a delayed transition from the formation of primary complexes to larger-sized aggregation. At the same time, increasing the charge density enhances electrostatic attraction to HA, which contributes to a higher growth rate once aggregation begins. This suggests that while high solubility delays the onset of floc growth, elevated charge density accelerates the subsequent aggregation process, highlighting the interplay between solubility and charge density in governing flocculation kinetics.

**Figure 8:**
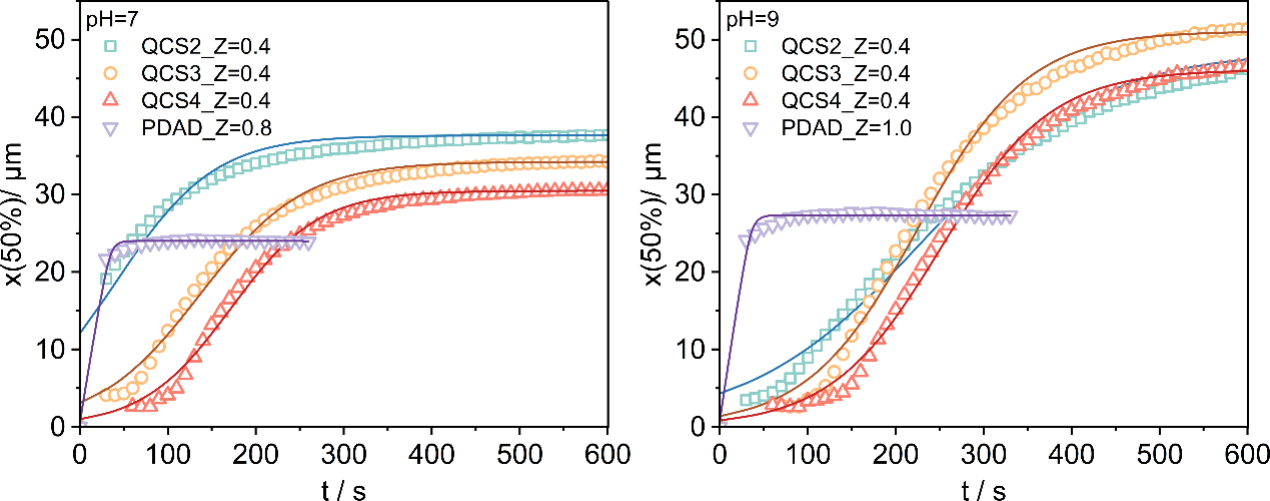
Median particle size (*x*(50%)) for complexes of HA and various QCSs with charge ratio *Z* = 0.4 at pH 7 and charge ratio *Z* = 0.5 at pH 9 as a function of time *t*. The trend diagrams of the median particle size (*x*(50%)) for HA-PDAD complexes with charge ratio *Z* = 0.8 at pH 7 and charge ratio *Z* = 1.0 at pH 9 are also included. Solid lines are fits to the logistic growth model.

Differences in the behavior of various QCS systems were also evident at a higher pH of 9.0 with charge ratio *Z* = 0.5, among which HA-QCS3 complexes exhibited the highest final size (*x*(50%)) of 51.1 μm, indicating the capacity of QCS3 to achieve larger flocs under similar pH conditions compared to the other modified chitosans. HA-QCS4 complexes showed the highest growth rate at 0.016 s^–1^ and the latest inflection point, similar to the situation at pH 7, suggesting a delayed but more rapid growth phase. Such delayed nucleation phenomena of HA complexes with higher charged QCS species could be attributed to their highest solubility, which on the other hand benefits the following flocculation process as their higher charge density prompts further interaction with HA molecules. Conversely, PDAD, which exhibits the highest charge density, forms with HA (see trend diagram in Figure S16) the smallest aggregates with a final size *x*(50%) of 24.0 μm with charge ratio *Z* = 0.8 at pH 7; similarly, HA-PDAD complexes with charge ratio *Z* = 1.0 at pH 9 exhibit a final *x*(50%) value of 27.3 μm. For both samples, the inflection points occurred at around 20 s, demonstrating the quickest flocculation kinetics. This rapid kinetics can be attributed to the high charge density of PDADMAC. However, the relatively small final floc size suggests that the strong solubility and relatively rigid backbone of PDADMAC may limit extensive polymer bridging compared to less highly charged or more hydrophobic QCS systems.

Additionally, the influence of pH on the flocculation dynamics of HA complexes with the same QCS at an identical charge ratio *Z* was investigated, and the corresponding change of *x*(50%) over time is shown in Figure S17. In general, in a more basic environment, larger aggregates are formed. For instance, the HA-QCS4 complexes exhibited a higher maximum *x*(50%) value of 46.1 μm at pH 9, in contrast to 39.1 μm at pH 7. However, there was a notable reversal in the inflection points for the different pH values. At pH 7, the time to reach half-maximal *x*(50%) was 80.9 s, whereas at pH 9, it extended significantly to 251.6 s. This phenomenon was also seen for HA-QCS2 complexes at charge ratio *Z* = 0.4, indicating that the flocculation process was significantly retarded by the higher pH.

## Conclusions

In this paper, we looked at the potential application of chitosan in water treatment by studying its ability to precipitate HA from aqueous solution as a model reaction for NOM removal. The solubility of chitosan at neutral and basic pH was enhanced by introducing permanent cationic charges, which was achieved by cationic modification of the amino group with GTMAC. The interaction of the differently modified chitosan (QCS) with negatively charged HA was systematically investigated in terms of phase diagrams and ζ-potential. For benchmarking, the commercial polycation PDAD was used as a reference. To correspond to the practical conditions in the water treatment process, measurements were performed at pH 7.0 and 9.0. In addition, it might be noted that the length and polydispersity of the two polymers do not naturally perfectly coincide but are quite similar, with a stretched length of the QCSs of 155–580 nm and that of PDADMAC being 310–620 nm.

Macroscopic observation shows that HA becomes precipitated when the addition of fully charged PDAD approaches the charge equilibrium. In contrast, such biphasic regions are seen for lower *Z* with QCSs, which can be explained by the QCS architecture, where the charged groups are connected to the polymer backbone via a spacer, thereby giving much higher flexibility to bind to the charged groups of the HA. Confocal microscopy showed that the formed HA flocs also varied with the charging state of the various QCSs. The biphasic region was shifted to lower *Z* at pH 7 compared to that at pH 9 due to the pH-dependence of the charging of both HA and QCS molecules. Fluorescence experiments with Prodan as solvatochromic dye showed that no significant formation of hydrophobic domains occurs during complexation, and only some effect is seen at higher pH, when the HA is fully charged.

The flocculation process of HA complexes was monitored through LLD measurements, which revealed distinct growth kinetics and particle size evolution of HA complexes formed with various QCSs and PDAD. Generally, one observes a rapid initial growth followed by a plateau phase of the size. The degree of permanent charging and pH significantly influence the flocculation kinetics, where the least charged QCS2 shows the fastest initial complexation, followed by the slowest subsequent growth rate among all QCSs, and ends up with the largest particle size at a pseudoequilibrium state. On the other hand, PDAD with the highest charge density shows the fastest flocculation kinetics but the smallest particle size in the plateau region. It should be noted that the molecular weights of QCS and PDADMAC are not exactly identical, although they fall within a comparable range (chitosan: 50–190 kDa; PDADMAC: 100–200 kDa). Therefore, we consider the comparison between QCS and PDADMAC in this study to be reasonable, with charge density and molecular architecture being the primary factors.

In summary, our findings underscore the critical role of cPE structure in tuning the precipitation and flocculation behavior of HA in aqueous solution. In general, it can be stated that QCS compares well to PDAD with respect to its performance in HA removal, with the advantage that one employs a biopolymer, and by modulating its chemical structure, one can tailor its performance correspondingly. Due to its architecture, it is able to lead to flocculation at a much lower charge ratio. Accordingly, our study should provide valuable insights into the design and optimization of flocculation processes in water treatment applications by the appropriate choice of a modified biopolymer.

## Supplementary Material



## Notes

Potentiometric titration curve of HA and chitosan; conductometric titration of modified chitosan; detailed description of UV254; and additional laser diffraction results (PDF)

## §

Innovative Water Services Solutions, Sydney, Australia
