# Supplementary material for: Application of Quaternized Chitosan in Enhancing Natural Organic Matter (NOM) Removal from Water by Flocculation
Source: Langmuir. 2026 Apr 8;42(15):10258–68. doi: 10.1021/acs.langmuir.5c06027 (PMC13104166; doi:10.1021/acs.langmuir.5c06027)
Supplement: Supplementary file 1 [file la5c06027_si_001.pdf]

Supporting Information

Application of Quaternised Chitosan in Enhancing NOM Removal from Water by Flocculation

Mingyu Yuan[1,], Heriberto Bustamante[2], Michael Gradzielski[1,*]*

1: Stranski-Laboratorium für Physikalische und Theoretische Chemie, Institut für Chemie, Technische Universität Berlin, D-10623 Berlin, Germany

2: Sydney Water, Parramatta NSW 2125, Australia

*Corresponding author: michael.gradzielski@tu-berlin.de; m.yuan.1@campus.tu-berlin.de

Number of Pages: 23

Number of Figures: 17

Characterization of IHSS (International Humic Substances Society)**-HA**

0.1 wt% IHSS HA was prepared with water and NaOH solution added on top, as humic acid is water soluble in its deprotonated state. Certain amount of HA solution was weighted on balance (ca. 15 g) and exact mass was noted before the conduction of titration measurements. The average molecular weight of the charged unit was determined using potentiometric titration with a 0.1 M HCl solution. From the titration curve, three inflection points were identified. The consumption of the acid solution between the first and second inflection points corresponds to the protonation of phenolic groups of the HA molecules, while the consumption between the second and third inflection points corresponds to the protonation of carboxylate anions. The titration curve was further transformed into the degree of dissociation as a function of pH by calculating the fraction of HCl consumed at a given pH relative to the total amount consumed between the first and third inflection points~~This titration curve then was also converted to the degree of dissociation as a function of pH~~, shown in Figure S1 right. The charge of humic acid can be calculated as 5.23 meq/g by the overall consumption between the first and the third inflection points. Considering the change of degree of dissolution along pH value, the average molecular weight of the charged unit at pH 9 is 255 g/mol, which can be further converted to the nominal charge concentration for 40 mg/L HA solution as 0.157 mM at pH 9.

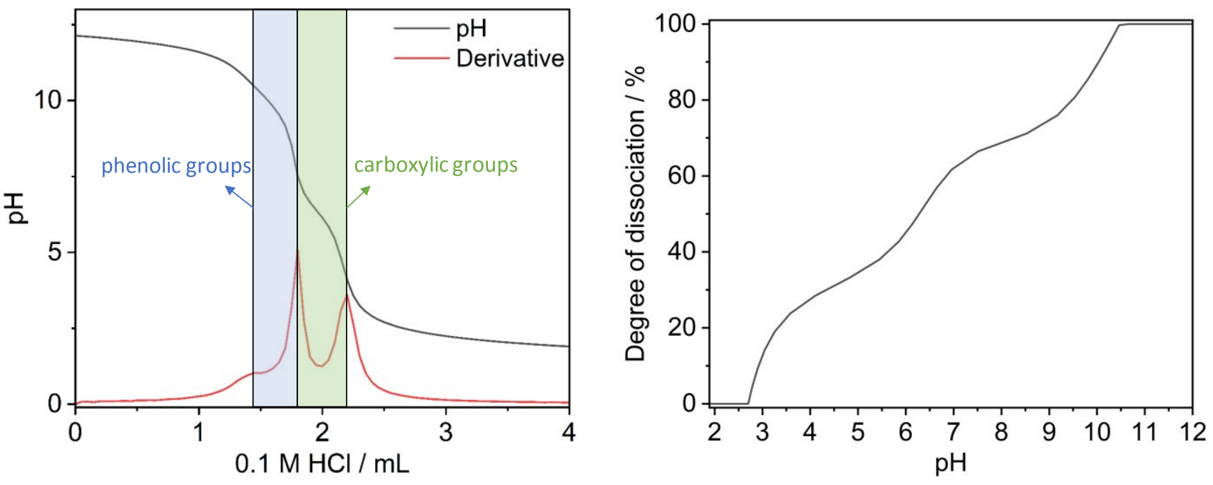

Figure S1. Potentiometric titration curve of humic acid with a solution of HCl and the corresponding degree of dissociation as a function of pH.

Degree of deacetylation (DDA) of chitosan

The degree of deacetylation (DDA) was determined by potentiometric titration with 0.1 M sodium hydroxide solution[1]. 1 wt% chitosan solution was prepared by dissolving chitosan in deionized water, followed by the addition of HCl to adjust the pH to approximately 4, ensuring complete solubilization of chitosan under acidic conditions. For the titration measurements, certain amount of CS solution was weighted on balance (ca. 10 g) and the exact mass was recorded prior to the titration experiments. From the titration curve in Figure S2 two inflexion points were derived, between which the consumption of basic solution indicating the deprotonation of ammonium cations from the chitosan. This parameter allows the calculation of the degree of deacetylation (DDA), which was determined to be 73%.

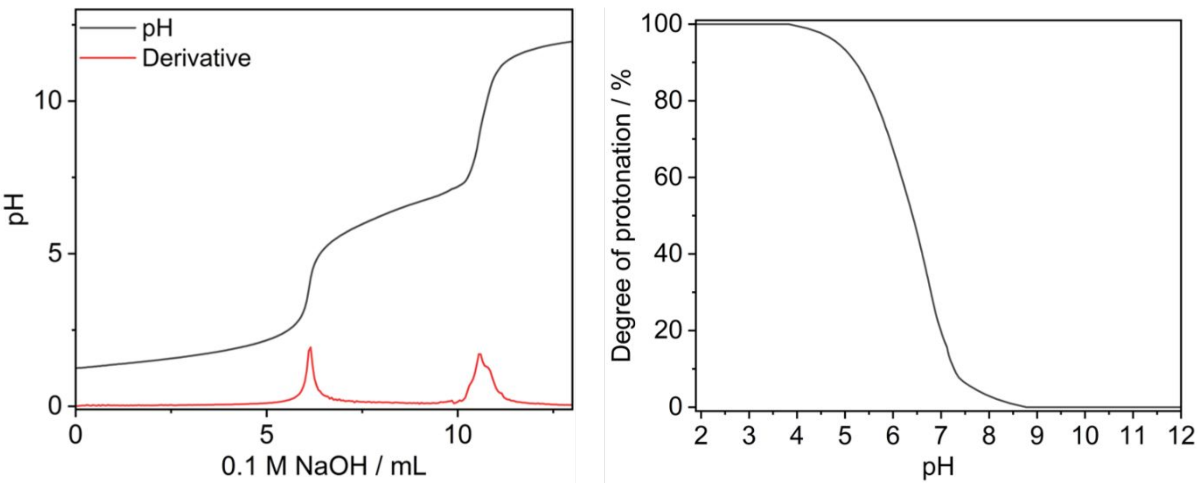

Figure S2. Determination of the degree of deacetylation (DDA) from potentiometric titration of chitosan.

QCSs characterization

The DS for each QCS variant was determined through a titration method reliant on conductivity measurements with the addition of 0.01 M $AgNO_3$[2]. 0.1 wt% QCS solution was dissolved in deionized water. 12 g of each QCS solutions was weighed on a balance prior to conducting the titration measurements. The formation of an insoluble salt between Ag^+ ions and the Cl^- (counterions of GTMAC) leads to an initial decrease in the conductivity of the QCS solutions, followed by an increase. The inflection point in this trend corresponds to the stoichiometric balance between the substituted GTMAC and the added Ag^+, thereby enabling the calculation of the substitution degree of GTMAC and of the permanent charge density of each QCS based on the average molecular weight of the substituted unit. The obtained values are summarized in Table 1.

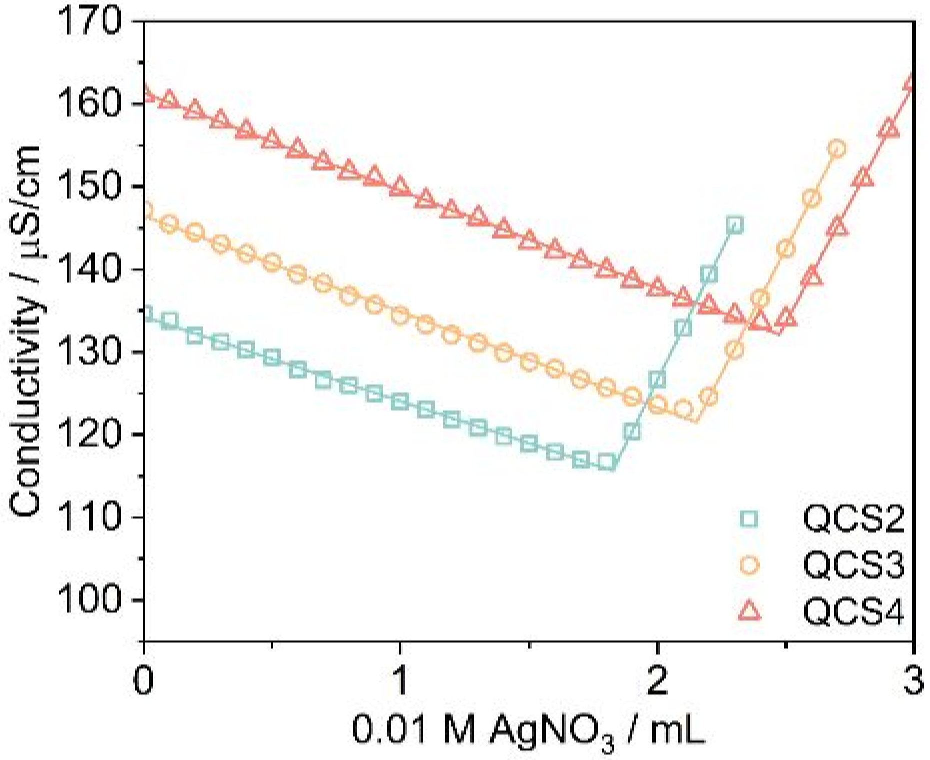

Figure S3. Determination of the degree of substitution (DS) of modified chitosan from conductometric titration with $AgNO_3$.

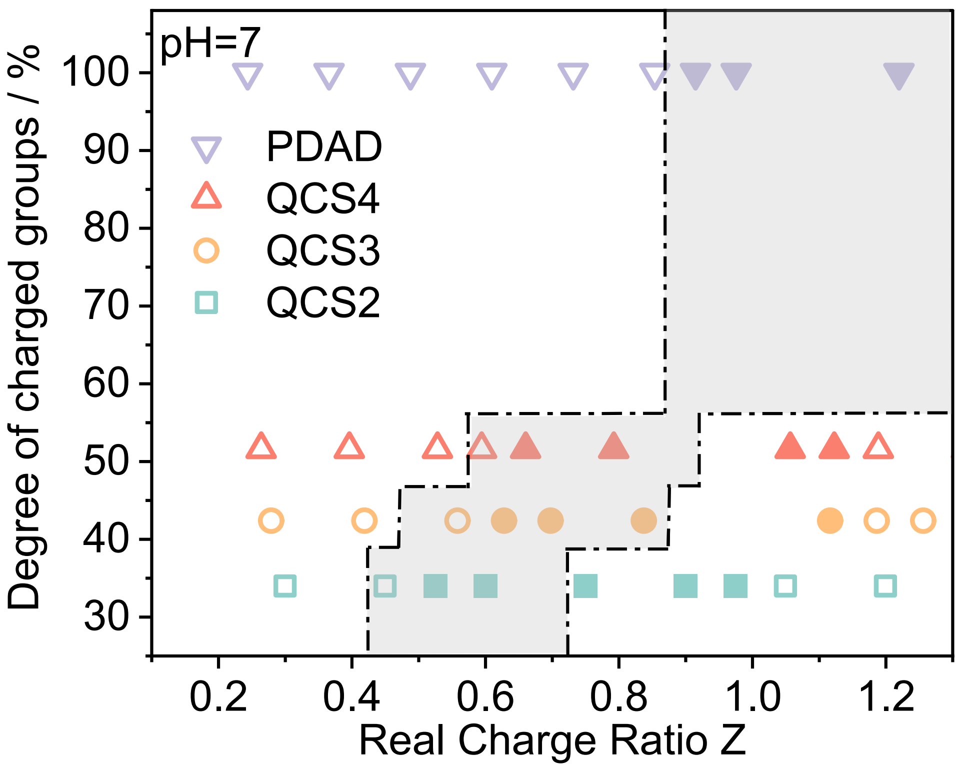

Figure S4. Phase diagram of 40 mg/L HA and added polyelectrolytes, the added amount being characterised by the real charge ratio Z (= [+]/[-]) at pH 7 (T = 25 ˚C). Open symbols refer to monophasic regions while full symbols refer to the biphasic region.

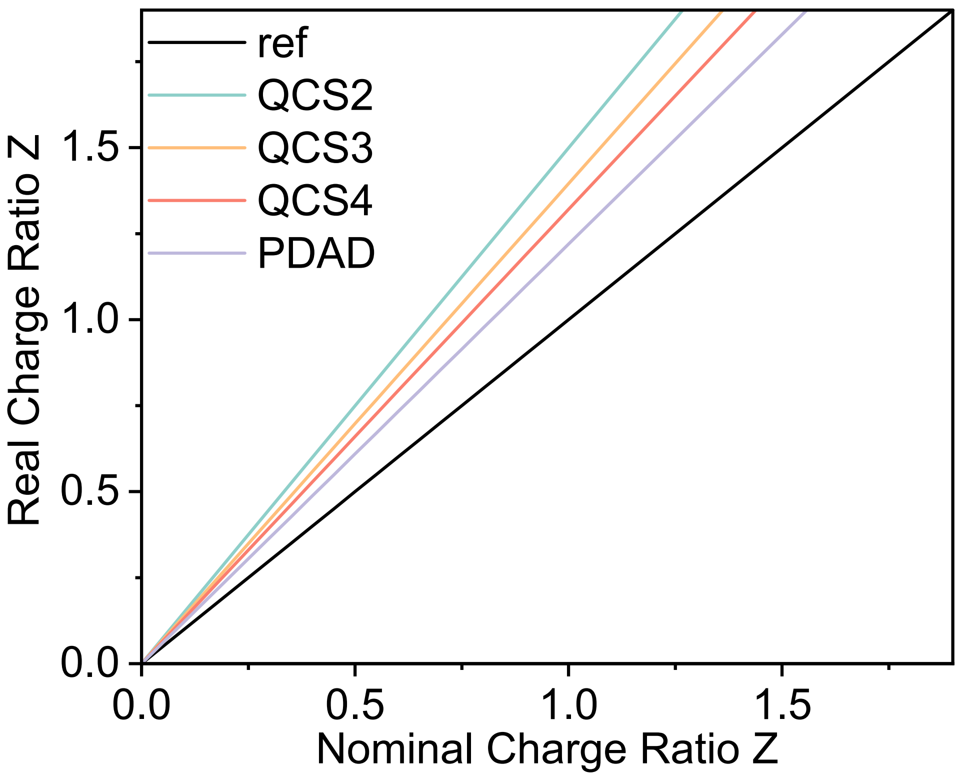

Figure S5. The relationship between the real charge ratio and the nominal charge ratio Z at pH 7. The calculation of the real charge ratio Z accounts for the actual ionization state of both the QCS and humic acid at pH 7. Specifically, for the QCS the degree of deprotonation of the residual amino groups was considered, as it varies depending on the degree of GTMAC substitution and thus differs among the three types of QCS used; for HA the protonation state was considered based on its dissolution curve as a function of pH. Together, these corrections allow for a more accurate representation of the effective charge ratio beyond the nominal value.

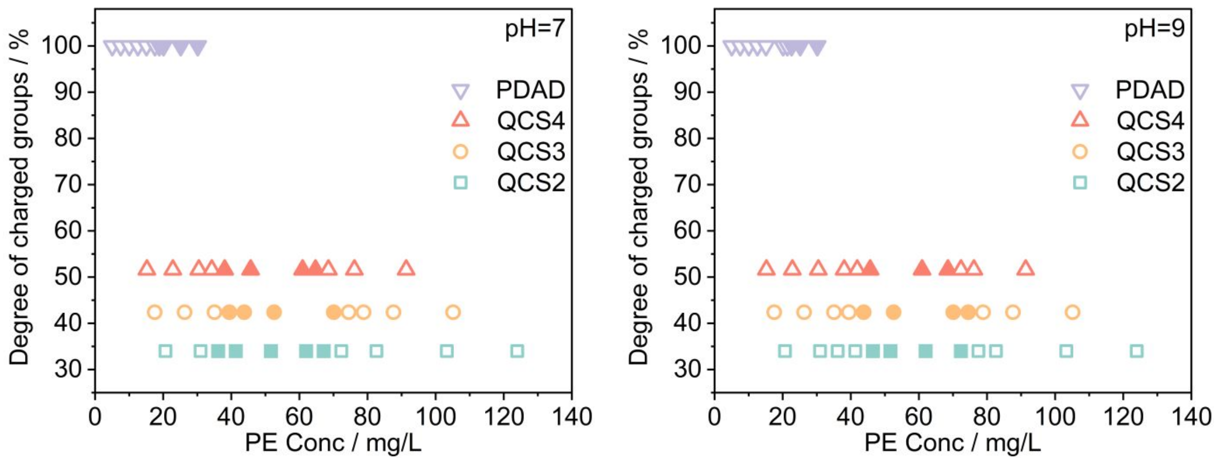

Figure S6. Phase diagram of 40 mg/L HA and added cationic polyelectrolytes, the added amount being characterised by the mass concentration of polyelectrolytes at pH 7 and pH 9, respectively (T = 25 ˚C). Open symbols refer to monophasic regions while full symbols refer to the biphasic region.

Table S1. ζ-potential for complexes of HA (40 mg/L) and different cationic polyelectrolytes, at pH 7 and pH 9, respectively, for different charge ratios Z (T = 25 ˚C).

Z	pH=7				pH=9			
	QCS2<br>ξ, mV	QCS3<br>ξ, mV	QCS4<br>ξ, mV	PDAD<br>ξ, mV	QCS2<br>ξ, mV	QCS3<br>ξ, mV	QCS4<br>ξ, mV	PDAD<br>ξ, mV
0	-26.2(1.5)				-33.0(1.6)			
0.2	-28.6(1.2)	-31.1(1.1)	-31.3(1.3)	-29.6(1.1)	-35.5(1.4)	-33.3(1.6)	-28.7(1.4)	-28.3(1.8)
0.3	-24.5(1.3)	-27.6(1.8)	-26.5(1.5)	-26.5(1.8)	-27.5(1.7)	-30.0(1.9)	-23.9(2.0)	-27.7(1.3)
0.4	-20.8(0.9)	-19.5(0.8)	-22.2(0.9)	-28.7(2.5)	-16.9(1.0)	-17.6(1.4)	-20.6(1.0)	-27.3(0.6)
0.5	-15.0(1.0)	-18.7(0.8)	-18.4(0.7)	-22.6(1.9)	-14.6(1.0)	-17.8(0.7)	-21.2(0.5)	-22.2(1.3)
0.6	-2.8(0.3)	-6.5(0.5)	-6.6(0.3)	-23.8(0.7)	2.1(0.3)	-9.5(0.5)	-13.0(0.4)	-21.5(0.4)
0.8	16.0(0.4)	11.9(0.2)	6.5(0.1)	-19.8(0.8)	7.0(0.4)	10.7(0.3)	5.2(0.4)	-20.1(0.7)
1	21.2(1.0)	19.4(1.1)	21.3(1.4)	-10.9(0.5)	17.3(0.6)	19.5(0.8)	16.8(0.5)	-13.2(0.9)
1.2	21.7(1.0)	24.2(1.1	24.4(0.7)	1.5(0.1)	19.7(0.8)	23.7(0.8)	24.1(0.8)	0.4(0.2)

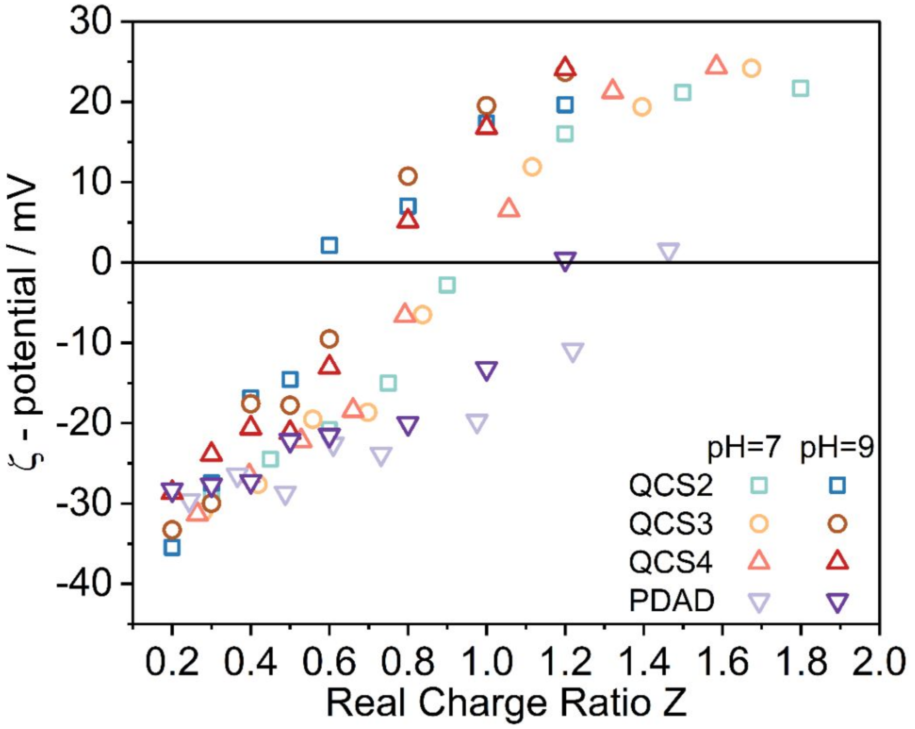

Figure S7. ζ-potential for complexes of HA and polyelectrolytes at different charge ratio Z at pH 7 and pH 9, respectively.

Removal efficiency of humic acid – UV-vis monitoring

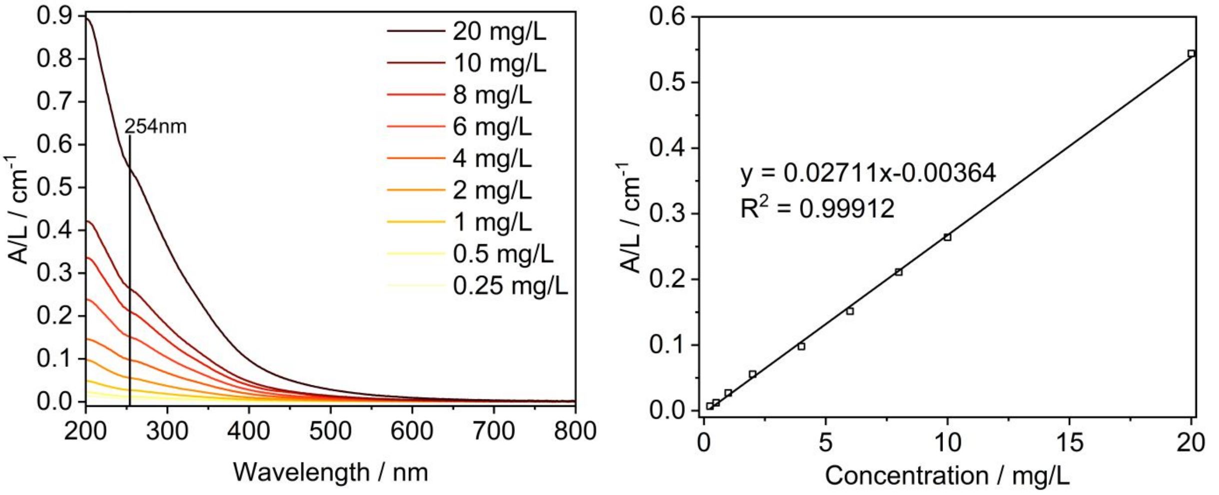

Figure S8. UV-vis spectra of humic acid solutions of different concentrations and the corresponding calibration curve for HA concentration as a function of UV absorbance at 254 nm.

Table S2. Decadic UV-absorbance at 254 nm per optical path length (UV254) for complexes of HA (40 mg/L) and different cationic polyelectrolytes, at pH 7 and pH 9, respectively, for different charge ratios Z (T = 25 ˚C).

	Z	A/L (cm-1)				HA conc (mg/L)				Remaining HA (%)			
		QCS2	QCS3	QCS4	PDAD	QCS2	QCS3	QCS4	PDAD	QCS2	QCS3	QCS4	PDAD
pH=7	**0.4**	0.26				9.8				24.4			
	**0.5**	0.18	0.26	0.41		7.0	9.9	15.1		17.4	24.8	37.7	
	**0.6**	0.18	0.27	0.18		6.9	10.2	7.0		17.2	25.5	17.4	
	**0.8**		0.48	0.18	0.31		17.7	6.7	11.5		44.3	16.7	28.8
	**1**				0.27				10.0				24.9
	**1.2**				0.17				6.6				16.4
pH=9	**0.4**												
	**0.5**	0.17	0.25			6.2	9.3			15.6	23.3		
	**0.6**	0.13	0.19	0.23		4.8	7.1	8.6		12.0	17.8	21.5	
	**0.8**		0.25	0.16			9.3	5.9			23.3	14.8	
	**1**				0.21				7.9				19.7
	**1.2**				0.11				4.3				10.8

*the supernatant of samples with biphasic region was measured 24 hours after mixing for different cationic polyelectrolytes at different charge ratio Z at 25 ˚C. The corresponding HA concentration and remaining HA percentage were calculated.

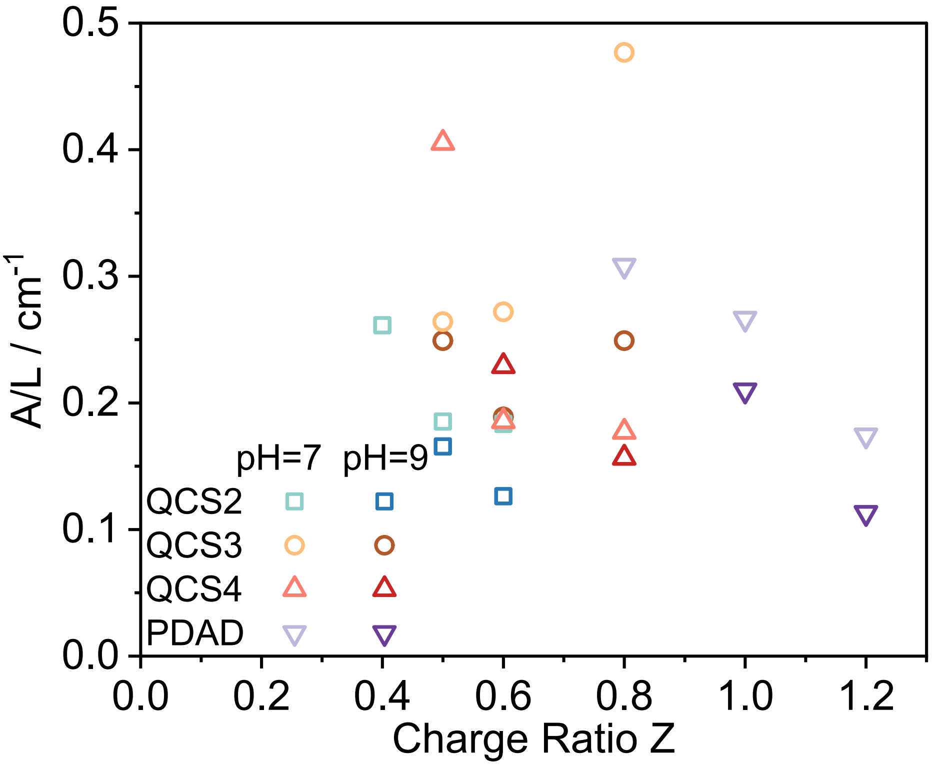

Figure S9. Decadic UV-absorbance at 254 nm per optical path length (UV254) of HA-PE systems (supernatant for biphasic region) 24 hours after mixing at pH 7 and pH 9, respectively, for different charge ratios Z at 25 ˚C.

Fluorescence Probe Studies

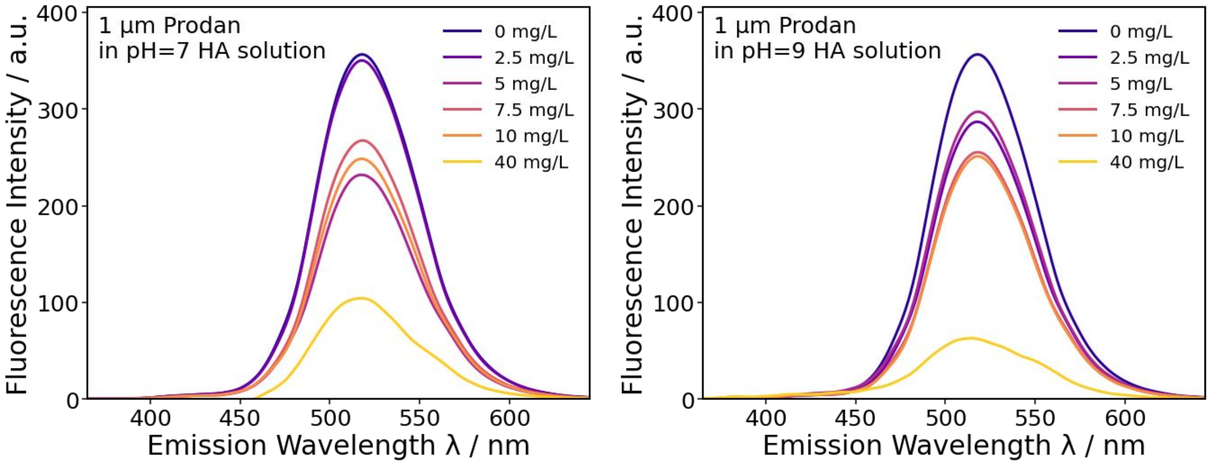

Figure S10. The fluorescence emission spectra of polarity-sensitive 1 µM Prodan probe in varying concentrations of humic acid at different pH.

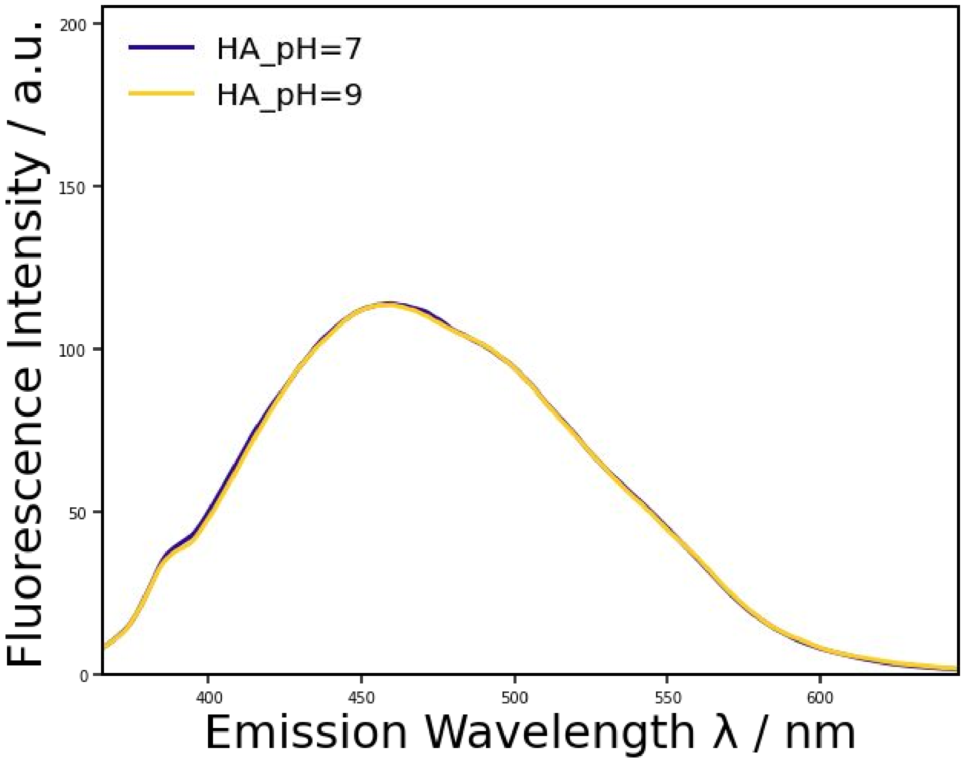

Figure S11. Fluorescence emission spectra of 40 mg/L humic acid at pH 7 and pH 9, respectively.

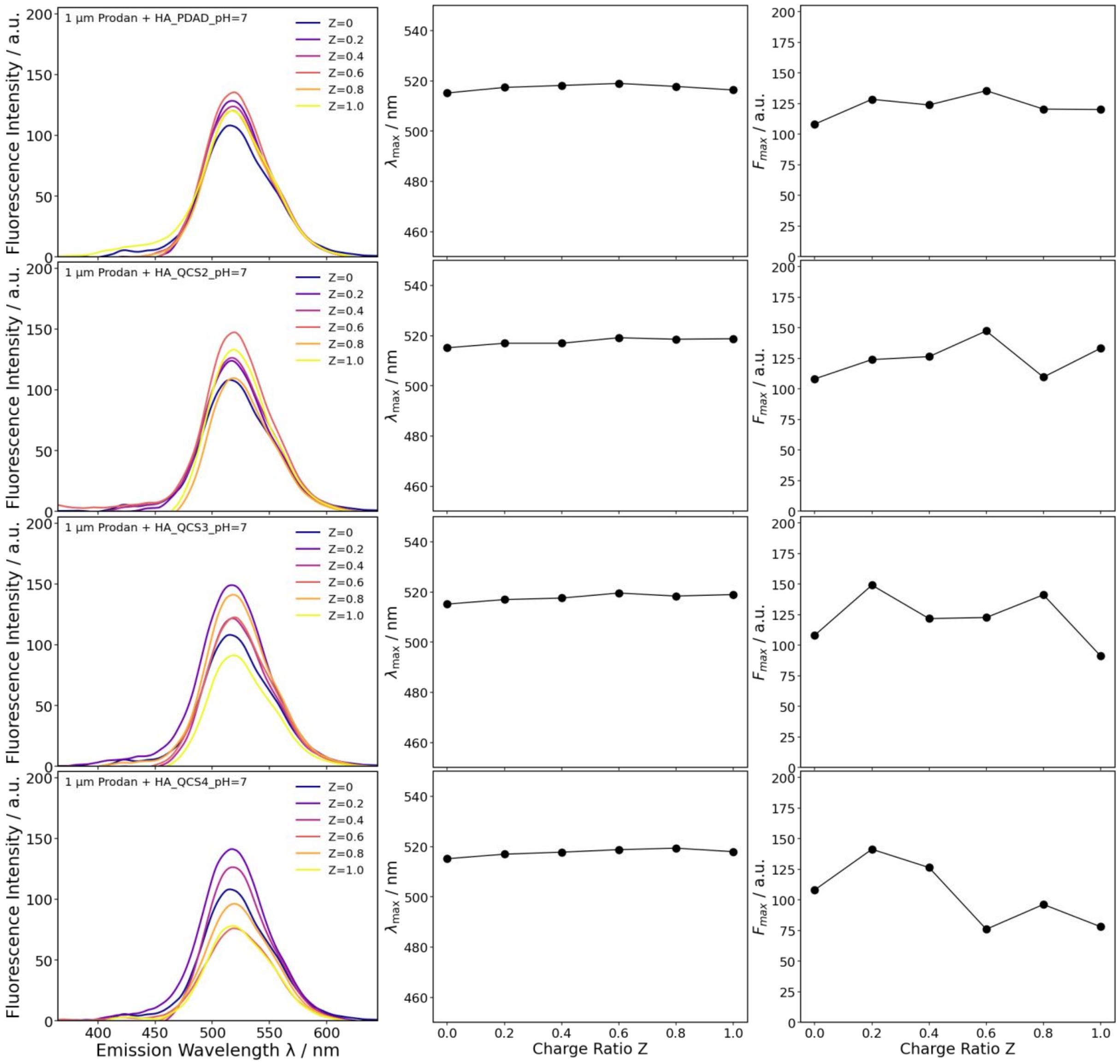

Figure 12 Fluorescence spectra of the various HA(40 mg/L)-cPE complexes containing 1 μM Prodan at pH 7 for different charge ratio Z at 25 ˚C and the extracted wavelength of the maximum emission (λ_{max}) and its emission intensity (F_{max}).

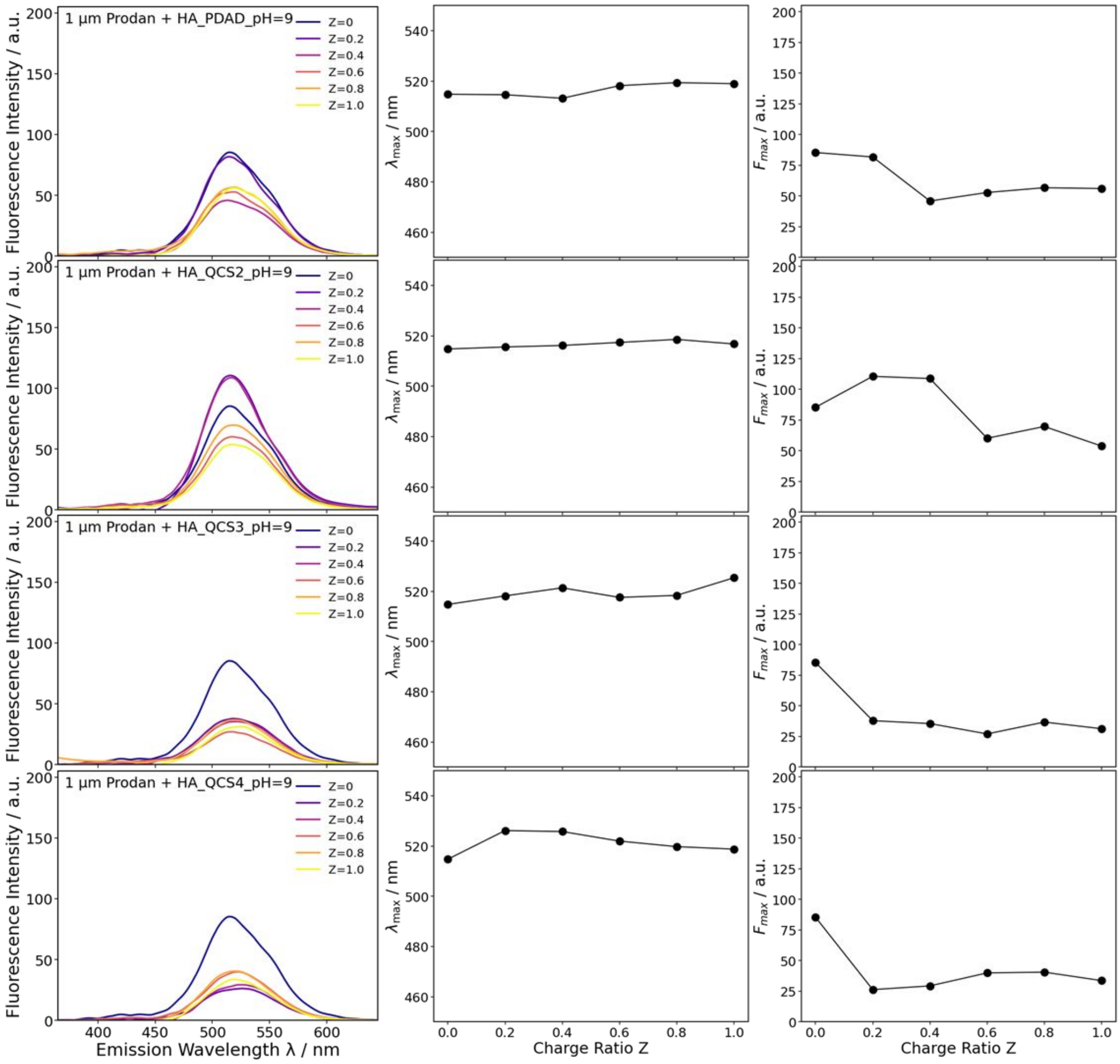

Figure S13. Fluorescence spectra of the various HA(40 mg/L)-cPE complexes containing 1 μM Prodan at pH 9 for different charge ratio Z at 25 ˚C and the extracted wavelength of the maximum emission (λ_{max}) and its emission intensity (F_{max}).

Flocculation of HA complexes via laser light diffraction

Two factors including size distribution density q3* and cumulative mass distribution Q_3 were utilized to elaborate the flocculation process of HA complexes. Specifically, size distribution density q_3^* represents the probability of finding a particle with a certain diameter within the population, whereas the cumulative mass distribution Q_3 is the integral curve for the size distribution, indicating the percentage of particles that are smaller than a specific diameter. The median percentiles of the particle size distribution, which are measured as the middle of cumulative mass distribution Q_3 curve, was denoted as x(50%). This parameter was adopted to provide a direct quantification of particle size during the flocculation process.

The change of the median percentiles of the particle size distribution x(50%) over time, characteristic of the size growth of HA complexes, was analysed with the logistic growth model, with

$$\mathrm{P(t)} = \frac{L}{1 + e^{-k(t-t_0)}}$$

Where P(t) is the particle size at time t, L is the maximum size the HA complexes can achieve, k is the growth rate and t_0 is the time at which the particle size is half of L.

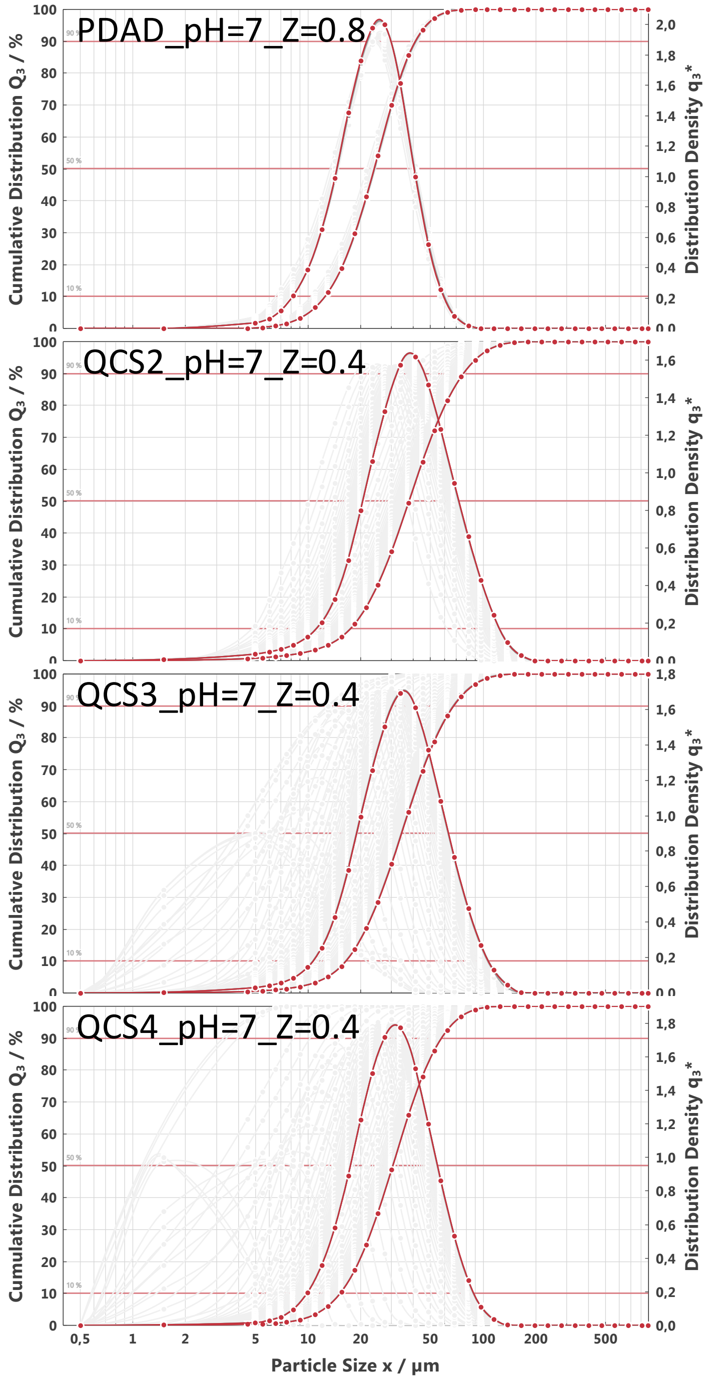

Figure S14. Laser diffraction particle size distribution density q_3^* and cumulative mass distribution Q_3 as a function of particle size recorded for complexes of HA and various QCSs with charge ratio Z=0.4 at pH 7 upon mixing. For HA-PDADMAC complexes, the charge ratio is 0.8. Each curve represents time interval of 10 s, in which the red one refers to the measurement at 300 s.

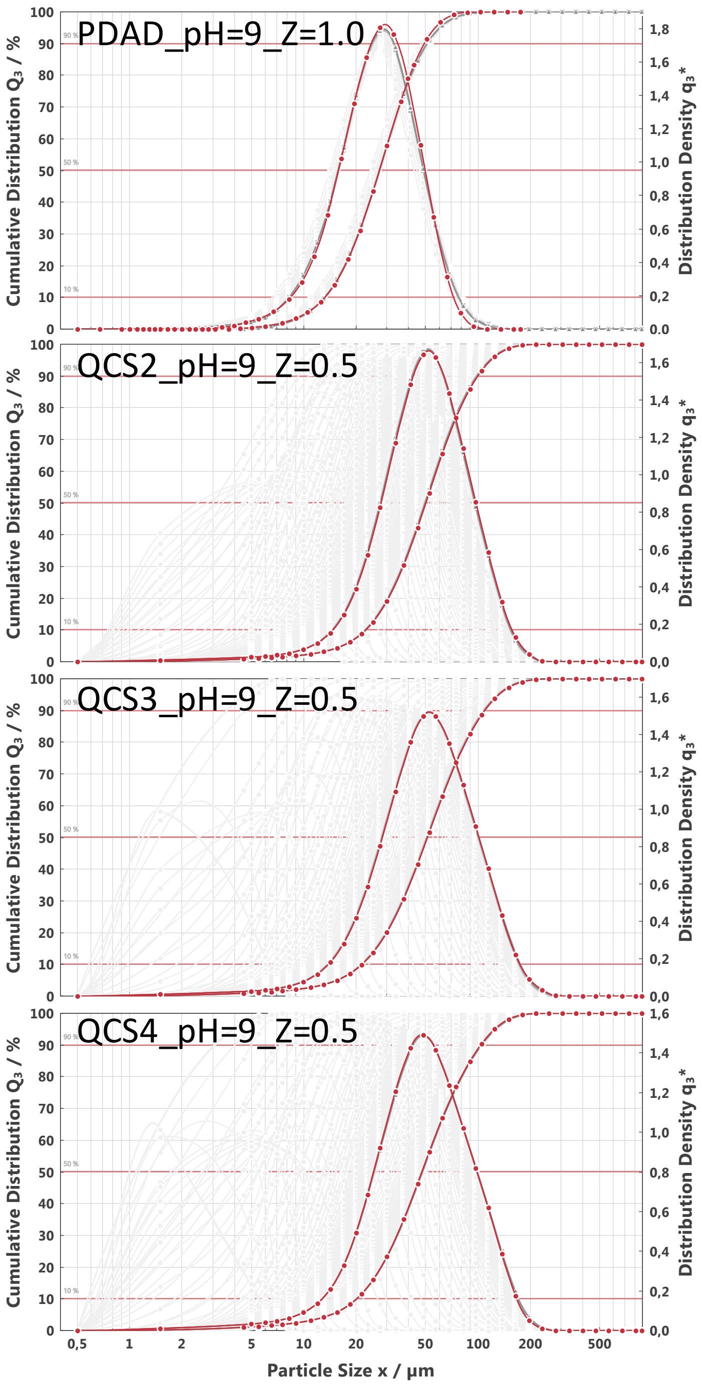

Figure S15. Laser diffraction particle size distribution density q_3^* and cumulative mass distribution Q_3 as a function of particle size recorded for complexes of HA and various QCSs with charge ratio Z=0.5 at pH 9. For HA-PDADMAC complexes, the charge ratio is 1.0. Each curve represents time interval of 10 s, in which the red one refers to the measurement at 300 s.

Table S3. The median size x(50%)$_M$ of the HA complexes, the growth rate k, the time t_0 at which the particle size is half of L and the corresponding goodness of fit R^2 as determined by the logistic growth model from the laser light diffraction data for complexes of HA and various cationic polyelectrolytes with various charge ratio Z.

		Z	L, µm	k, s^{-1}	t_0, s	R^2
pH=7	QCS2	0.4	37.65	0.018	41.89	0.9068
	QCS3	0.4	34.24	0.017	136.90	0.9897
	QCS4	0.4	30.49	0.020	171.39	0.9921
		0.5	39.05	0.014	80.85	0.9698
	PDAD	0.8	24.01	0.199	19.62	0.9921
pH=9	QCS2	0.4	46.77	0.019	164.15	0.9897
		0.5	48.71	0.010	233.81	0.9909
	QCS3	0.5	51.12	0.016	225.02	0.9926
	QCS4	0.5	46.14	0.016	251.18	0.9959
		0.6	65.12	0.005	444.19	0.9861
	PDAD	1.0	27.33	0.178	19.87	0.9865

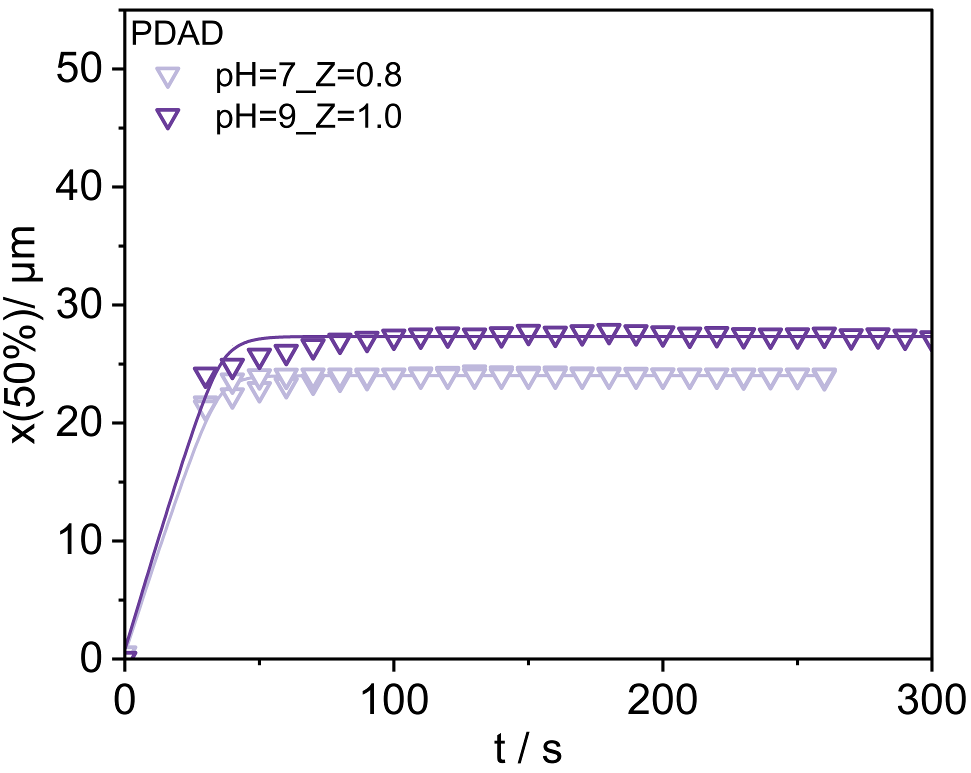

Figure S16. The trend diagram of the median particle size (x(50%)) for HA-PDADMAC complexes with charge ratio Z=0.8 at pH 7 and charge ratio Z=1.0 at pH 9. Solid lines are fits with the logistic growth model. Given the fact that here one sees only the end of the process, the parameters deduced from the fit have little robust meaning.

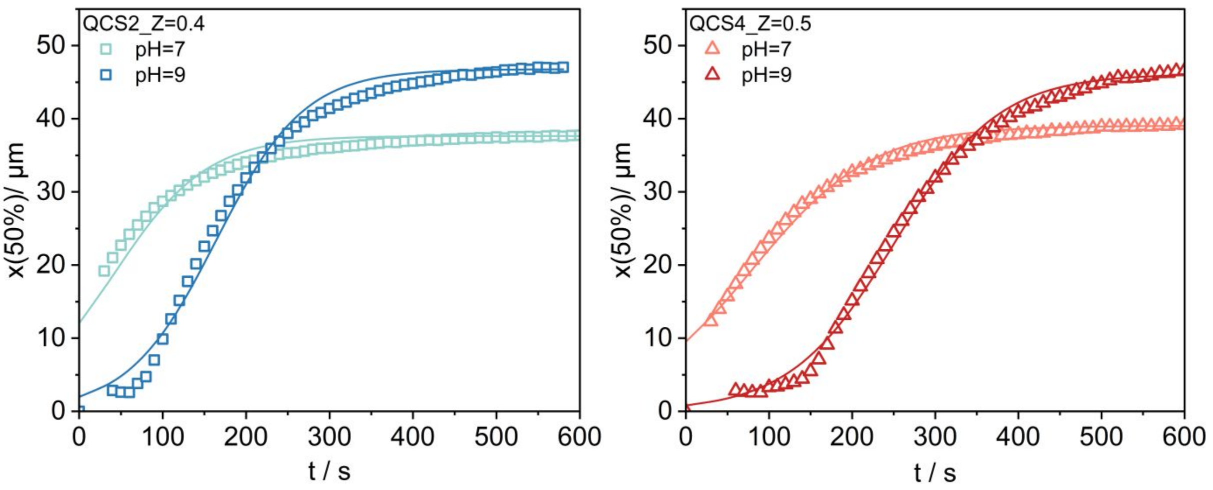

Figure S17. The trend diagram of the median particle size (x(50%)) for HA-QCS2 complexes with charge ratio Z=0.4 and HA-QCS4 complexes with charge ratio Z=0.5 under various pH conditions.

REFERENCES

1. Tolaimate, A.; Desbrieres, J.; Rhazi, M.; Alagui, A.; Vincendon, M.; Vottero, P., On the influence of deacetylation process on the physicochemical characteristics of chitosan from squid chitin. *Polymer* **2000,** *41* (7), 2463-2469.

2. Cho, J.; Grant, J.; Piquette-Miller, M.; Allen, C., Synthesis and physicochemical and dynamic mechanical properties of a water-soluble chitosan derivative as a biomaterial. *Biomacromolecules* **2006,** *7* (10), 2845-2855.
